# Circulating cell-free DNA-based epigenetic assay can detect early breast cancer

**DOI:** 10.1186/s13058-016-0788-z

**Published:** 2016-12-19

**Authors:** Natsue Uehiro, Fumiaki Sato, Fengling Pu, Sunao Tanaka, Masahiro Kawashima, Kosuke Kawaguchi, Masahiro Sugimoto, Shigehira Saji, Masakazu Toi

**Affiliations:** 1 Department of Breast Surgery, Graduate School of Medicine, Kyoto University, Kyoto, Japan; 2 Department of Target Therapy Oncology, Graduate School of Medicine, Kyoto University, Kyoto, Japan; 3 Institute for Advanced Biosciences, Keio University, Tsuruoka, Yamagata Japan; 4 Department of Medical Oncology, Fukushima Medical University, Fukushima, Japan

**Keywords:** Circulating DNA, Breast cancer, Epigenetics, DNA methylation, Early detection

## Abstract

**Background:**

Circulating cell-free DNA (cfDNA) has recently been recognized as a resource for biomarkers of cancer progression, treatment response, and drug resistance. However, few have demonstrated the usefulness of cfDNA for early detection of cancer. Although aberrant DNA methylation in cfDNA has been reported for more than a decade, its diagnostic accuracy remains unsatisfactory for cancer screening. Thus, the aim of the present study was to develop a highly sensitive cfDNA-based system for detection of primary breast cancer (BC) using epigenetic biomarkers and digital PCR technology.

**Methods:**

Array-based genome-wide DNA methylation analysis was performed using 56 microdissected breast tissue specimens, 34 cell lines, and 29 blood samples from healthy volunteers (HVs). Epigenetic markers for BC detection were selected, and a droplet digital methylation-specific PCR (ddMSP) panel with the selected markers was established. The detection model was constructed by support vector machine and evaluated using cfDNA samples.

**Results:**

The methylation array analysis identified 12 novel epigenetic markers (*JAK3*, *RASGRF1*, *CPXM1*, *SHF*, *DNM3*, *CAV2*, *HOXA10*, *B3GNT5*, *ST3GAL6*, *DACH1*, *P2RX3*, and chr8:23572595) for detecting BC. We also selected four internal control markers (*CREM*, *GLYATL3*, *ELMOD3*, and *KLF9*) that were identified as infrequently altered genes using a public database. A ddMSP panel using these 16 markers was developed and detection models were constructed with a training dataset containing cfDNA samples from 80 HVs and 87 cancer patients. The best detection model adopted four methylation markers (*RASGRF1*, *CPXM1*, *HOXA10*, and *DACH1*) and two parameters (cfDNA concentration and the mean of 12 methylation markers), and, and was validated in an independent dataset of 53 HVs and 58 BC patients. The area under the receiver operating characteristic curve for cancer-normal discrimination was 0.916 and 0.876 in the training and validation dataset, respectively. The sensitivity and the specificity of the model was 0.862 (stages 0-I 0.846, IIA 0.862, IIB-III 0.818, metastatic BC 0.935) and 0.827, respectively.

**Conclusion:**

Our epigenetic-marker-based system distinguished BC patients from HVs with high accuracy. As detection of early BC using this system was comparable with that of mammography screening, this system would be beneficial as an optional method of screening for BC.

**Electronic supplementary material:**

The online version of this article (doi:10.1186/s13058-016-0788-z) contains supplementary material, which is available to authorized users.

## Background

Breast cancer (BC) is the most prevalent cancer and the leading cause of cancer deaths in women all over the world [1]. Currently, mammography is the standard method for early detection of BC in many countries. However, false-positive recall rates vary according to age, breast density, and postmenopausal hormonal therapy, among others [2, 3]. For women with dense breasts, the accuracy of mammography is decreased. As the breast density of Asian women is relatively high [4], there is an unmet need for the development of accurate BC screening methods. It is reported that ultrasonography helps to improve the sensitivity of detection in young Japanese women; however, there are some technical hurdles for standardization [5].

Blood-based methods for monitoring of BC have been in development for several decades. Conventional tumor markers, such as carcinoembryonic antigen (CEA), cancer antigen (CA)15-3 [6–8], and circulating tumor cell (CTC) count [9], are clinically available. However, their usefulness is mostly limited to patients with advanced and metastatic BC (MBC). Recently, circulating cell-free DNA (cfDNA) has received considerable attention as a resource of cancer biomarkers. Dawson et al. demonstrated that cfDNA-based markers (cancer-derived gene mutations) were more useful for monitoring metastatic BC than conventional tumor markers and CTC count [10]. As the cfDNA is thought to contain DNA derived from tumor cells in the whole body, tumor evolution can be also monitored by profiling the DNA mutation pattern.

Somatic gene mutations are highly specific events in cancer and precancerous lesions that can be useful in detecting cancer using remote samples. Technological approaches to quantifying tiny amounts of mutated DNA have been developed, such as digital PCR and barcode next-generation sequencing. However, in terms of cancer screening, next-generation sequencing is too expensive, and has a throughput capacity that is too low to process a large number of samples. In addition, detecting unknown mutated genes in cfDNA by a PCR-based method is difficult because mutation sites vary, even in highly mutated genes.

DNA methylation is an epigenetic system that regulates gene expression, and aberrant DNA methylation is associated with various pathologic events, including tumorigenesis and aggressive phenotypes of cancer. Since Silva et al. detected a methylated DNA fragment of the p16 promoter region in plasma samples from patients with BC [11], many reports have shown aberrantly-methylated DNA in plasma and serum [12–20]. However, the detection rates of these DNA methylation markers in the blood are low even in cases of advanced disease, and are therefore inadequate for early detection of BC [12, 13, 16–18]. In the present study, we aimed to develop a highly sensitive cfDNA-based system for early detection of BC using epigenetic biomarkers and digital PCR technology.

## Methods

Detailed information on the materials and methods used in this study is provided in Additional file 1.

### Cell culture

The cell lines used in this study are listed in Additional file 2: Table S1. Cells were grown according to the distributors’ recommended conditions.

### Collection of clinical samples

All blood and tissue samples were provided from a multi-institutional biobank project, the Breast Oncology Research Network (BORN)-Biobank, which was initiated and is maintained by the Department of Breast Surgery, Kyoto University. Blood samples from patients with BC were obtained after they received a traditional diagnosis of BC. In this study, BC stage 0-I was considered early BC.

### Laser capture microdissection (LMD) of BC tissue specimens

Individual 10-μm-thick formalin-fixed paraffin-embedded (FFPE) specimens of surgically resected BC tissue were placed on Leica foil membrane slides, and immunohistochemically stained by pan-cytokeratin antibody cocktails (AE1/AE3, Dako, Glostrup, Denmark, M3515). Histo/Zyme (Diagnostic BioSystems, Pleasanton, CA, USA; DBS-K046-15) was used for antigen retrieval, and VECTOR Red Alkaline Phosphatase Substrate Kit (VECTOR Laboratories, Burlingame, CA, USA; SK-5100) was used for visualization. LMD of the stained FFPE slides was performed using LMD7000 systems (Leica microsystems, Wetzlar, Gemany). Cancer cell clusters from the BC samples were selectively microdissected (Additional file 3: Figure S1). Normal samples obtained from adjacent normal mammary epithelia and intraductal papilloma epithelia were also microdissected. Adjacent normal epithelia from 10 patients were pooled as a single sample.

### Comprehensive DNA methylation profiling

Using an Illumina Infinium Human Methylation 450 BeadChip Assay (Illumina, San Diego, CA, USA), we conducted comprehensive DNA methylation profiling of 56 laser-microdissected FFPE samples (38 luminal, 4 luminal human epidermal growth factor receptor 2 (HER2), 1 HER2, and 11 triple-negative (TN) types of BC, one pooled normal epithelia sample, and one intraductal papilloma sample), 34 samples of DNA from 31 cultured cells (4 luminal, 3 luminal HER2, 2 HER2, and 18 TN types of BC, 1 unknown type of BC, and 3 non-BC cells), and 29 white blood cell DNA samples from healthy volunteers (HVs), as listed in Additional file 2: Tables S1-S3. The peak bias in β-values of the two different probe types was corrected by an NIMBL toolbox [21] for MATLAB software.

At the selection of candidate markers, we attached importance to the difference of the methylation patterns based on the BC subtypes. To build a generalized multi-marker mathematical model for BC detection and avoid over-fitting, it is important to use several types of variables. Thus, we decided to select candidate markers from subtype-specific methylation loci, not only from loci commonly methylated in BC.

The mean β-values of the non-BC samples (meanNC), all BC samples (meanBC), and the luminal-type (meanLum) and TN-type (meanTN) of BC samples, were calculated. We selected the candidate markers from array probes with meanNC <0.05. The additional selection conditions of the candidate markers were as follows; (a) top 20 loci of the widest gap between meanBC and meanNC; (b) top 20 loci of the lowest meanNC with meanBC >0.6; (c) top 50 loci with the largest values of meanLum – meanTN; and (d) top 50 loci with the largest values of meanTN – meanLum. We referred to (a) and (b) as common BC markers, (c) as luminal-dominant markers, and (d) as TN-dominant markers. As the proportions of the cell lines and FFPE samples were different in the luminal and TN samples, direct calculation of the mean by sample type would be biased. To avoid such a bias, the mean β-values of each group were calculated as an average of the mean of the cell line samples and the mean of the FFPE samples. To evaluate the statistical significance of these markers, we calculated the *p* values using the Welch *t* test (Additional file 2: Table S4).

### Screening of DNA methylation markers using real-time quantitative methylation-specific PCR (MSP)

We used the Taqman-based MSP method in this screening step. To save screening costs and time, we utilized the Universal Probe Library (UPL, Roche Diagnostics GmbH, Mannheim, Germany) to design Taqman-MSP primers and probes. As the sequence variety of UPLs is limited, we designed primers and probes as close as possible to the candidate loci selected by the methylation array analysis (Additional file 2: Table S4). The MSP reaction mix consisted of 10 μl of FastStart Universal Probe Master (ROX) (Roche Diagnostics GmbH), 1 μl of primer mix for MSP (finally 0.5 μM), 0.4 μl of UPL probe, 2 μl of template bisulfite-treated DNA, and H_2_O up to 20 μl in total. The PCR reaction was performed using the StepOnePlus Real-Time PCR System (Applied Biosystems, Foster City, CA, USA) as follows; one cycle at 95 °C for 10 minutes, fifty cycles at 95 °C for 15 sec and 60 °C for 1 minute. A standard curve was generated using serially diluted, fully methylated DNA synthesized by SssI methyltransferase (New England Biolabs, Ipswich, MA, USA), and methylation values were normalized by MSP values of the *ACTB* gene as previously described [22].

Primers were selected on the basis of the following: (1) the efficiency of MSP was >70% and <110%; (2) methylation was detected in one or none of the samples of blood DNA from HVs; (3) methylation was detected in more than one sample of the DNA from the cultured cell lines; (4) methylation was not detected in the DNA derived from FFPE samples of adjacent normal epithelia; and (5) expression of the related genes was regulated by DNA methylation.

### Validation of candidate DNA methylation markers with the public database

To evaluate the universality of candidate markers, we analyzed the methylation data of peripheral blood mononuclear cells (PBMC, GSE58888) [23], and BC in The Cancer Genome Atlas (TCGA) Project [24] generated by the TCGA Research Network (http://cancergenome.nih.gov/). Then we showed the methylation pattern of samples with candidate markers in a heat map format. The distributions of the β-values for the selected methylation markers were compared among PBMC samples, all cancer samples, luminal BC samples, and basal-like BC samples using the Welch *t* test.

### Pharmacological unmasking of epigenetically silenced genes

To determine whether the expression of the screened marker genes was epigenetically regulated, MCF7, T47D, MDA-MB-231, and Hs578T were treated with the demethylating agent 5’-Aza-2-deoxycytidine (5’-Aza-dC) (Sigma-Aldrich, St. Louis, MO, USA) at 1 μM for 48 hours, and both 5’-Aza-dC and histone deacetylase inhibitor trichostatin A (Sigma-Aldrich) at 300 nM for 24 hours. DNA and RNA samples were then extracted. The methylation status of each selected marker was measured by quantitative MSP, as described. The RNA expression level of each gene was assessed by one-step reverse transcription PCR (RT-PCR) using a QuantiTect Probe RT-PCR Master Mix (QIAGEN, Venlo, Netherlands) according to the manufacturer’s protocol.

### Establishing the MSP assay using droplet digital PCR

To quantify tiny amounts of methylated DNA in cfDNA, we employed droplet digital PCR. To adjust selected primer/probe sets to duplex droplet digital PCR format, custom dual-labeled locked nucleic acid probes with FAM or Alexa Fluor® 532 dye and Black hole-1 quencher were synthesized for certain markers (Gene Design Inc., Ibaraki, Osaka, Japan). The final sequences of MSP primers and probes for selected markers are listed in Additional file 2: Table S5.

In epigenetic research, a primer/probe set developed by Eads et al., which targets the upstream region of *ACTB* [22], was traditionally used as an internal control reaction of MSP and also used in marker screening steps. However, in this study, the amplification efficiency of this primer/probe set was not sufficient. In addition, because the amounts of loaded cfDNA samples in droplet digital methylation-specific PCR (ddMSP) reactions are unknown and considerably varied, precise quantification is very important in an assay detection system. Therefore, we developed a panel of four novel internal control markers. We selected four internal control genes of which the copy number alteration ratios were less than 5%, according to the cBioPortal database (http://cbioportal.org) [25, 26]. The primer/probe sets for internal control markers were designed to target genomic regions containing no CpG, in order to amplify the region regardless of methylation status (Additional file 2: Table S6).

### Detecting methylated DNA markers in cfDNA by ddMSP

The extraction of cfDNA from plasma was conducted using QIAmp Circulating Nucleic Acid Kit (QIAGEN) with a modification of the manufacturer’s protocol to improve the cfDNA yield. Briefly, 900 μl of thawed plasma was mixed with 100 μl of PBS, 800 μl of Buffer ACL (lysis buffer), and 100 μl of proteinase K solution, and then was incubated at 48 °C for 18 hours with shaking. The sample was then mixed with an additional 100 μl of proteinase K solution by pulse-vortexing for 30 seconds, and was incubated for a further 6 hours. Finally, approximately 20 μl of cfDNA solution was eluted.

Following the manufacturer’s protocol, duplex ddMSP reactions were performed in a T100 thermal cycler (Bio-Rad, Hercules, CA, USA), and droplet signals were quantified by a QX100™ Droplet Reader (Bio-Rad). In total, 278 cfDNA samples from 145 patients with BC and 133 HVs were analyzed using this ddMSP assay, and all raw droplet signal data were exported from the built-in software, and manually analyzed using MATLAB software as follows.

### Data analysis of ddMSP data and development of the detection model

First, a sample dataset of 278 cases was randomly divided into a training set (*n* = 167) and a validation set (*n* = 111), each set being in accordance with the proportion of cancer patients and HVs, and with BC stage. Clinicopathological characteristics of the patients for cfDNA are shown in Table 1 and Additional file 2: Table S7. A detection algorithm was developed using the training dataset only. For each marker, optimized lower and upper cutoff thresholds for droplet amplitude were determined to maximize the area under the curve (AUC) of the receiver operator characteristic (ROC) curve as a single marker. The concentration of the methylated marker DNA fragments (copies/ml) was then calculated for each sample. The cutoff concentration for each marker was determined to divide the samples into marker-negative and marker-positive groups. All marker concentration values were converted into log10 values. Thus, the whole training dataset consisted of a total of 15 variables, including the concentration values of 12 DNA methylation markers and their mean value, a mean of four internal control markers, and the number of methylation-positive markers.

**Table 1:** Characteristics of healthy volunteers and patients with breast cancer

		Whole set (*n* = 278)	Training set (*n* = 167)	Validation set (*n* = 111)
Number of samples	HVs	133	80	53
	Patients with BC	145	87	58
Mean age (range)	HVs	45.3 (22–70)	45.8 (26–70)	44.5 (22–66)
	Patients with BC	59.5 (36–81)	59.8 (36–81)	59.1 (36–81)
Subtype	Luminal	98	58	40
	Triple-negative	25	14	11
	HER2	10	6	4
	Luminal HER2	8	6	2
	not assessed (DCIS)	4	3	1
Stage	0	4	3	1
	I	47	27	20
	IIA	31	19	12
	IIB	22	11	11
	III	9	8	1
	IV	32	19	13
Early BC (Stage0-I)	Luminal	36	22	14
	Triple-negative	8	3	5
	HER2	2	2	0
	Luminal HER2	1	0	1
	not assessed (DCIS)	4	3	1

*HVs* healthy volunteers, *BC* breast cancer, *HER2* human epidermal growth factor receptor 2, *DCIS* ductal carcinoma in situ, *Subtype* immunohistochemically categorized subtype

We developed a BC detecting model using a support vector machine (SVM) to distinguish patients with cancer from HVs. To determine the best variable set for the model, we tested all of the variable combinations (*n* = 2^15^ − 1). For each combination, the detection accuracy was estimated by leave-one-out cross-validation (LOOCV). The model that achieved the best AUC and coefficients of each variable that were >0, was then selected as the detection model.

To validate the robustness of the selected model, an independent dataset was used. The validation dataset was prepared using thresholds of droplet signals and cutoffs for marker concentration determined by the training dataset. The best SVM model selected above was applied to the validation data set. The accuracy of the detection model for the validation set was assessed using the AUC. Furthermore, we also performed ROC analysis and calculated the AUC to evaluate the performance of the model within each stage of BC as a subgroup analysis.

### Statistical analysis

Methylation assay analysis, processing of ddMSP data, and algorithm construction were performed using MATLAB software. Statistical analyses, such as correlation analysis, tendency analysis, and *t* statistics, among others, were performed using R software.

## Results

### Comprehensive DNA methylation array analysis

**Fig. 1:**
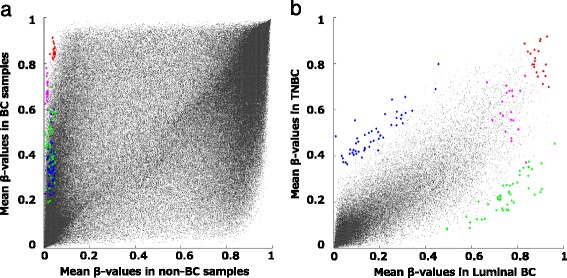
Methylation array data in a scatter plot format. *Colored dots* represent 140 candidate loci selected according to methylation array analysis. **a** Distribution of the mean β-values of the non-breast cancer (*BC*) samples (*meanNC*) and of the BC samples (*meanBC*) in the whole of the array probes (*n* = 482,421). All selected candidate markers had <0.05 of meanNC. **b** Distribution of mean β-values of luminal BC (*meanLum*) and of triple-negative BC (*meanTN*) in array probes with meanNC <0.05 (*n* = 121,079). *Red dots* common BC markers selected by condition (**a**). *Magenta dots* common BC markers selected by condition (**b**). *Green dots* luminal-dominant markers. *Blue dots* TN-dominant markers

**Fig. 2:**
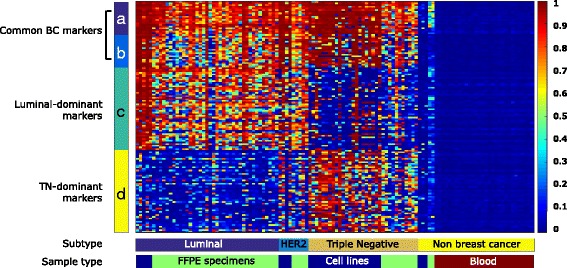
Heat map of 140 selected candidate markers. In the *heat map*, the *color* of each *square* represents the methylation level (ß-value) by methylation array, as a *color scale bar* (*right*) indicates. The *ribbons* (**a**-**d**) indicate the selectiong conditions described in “Methods”. *BC* breast cancer, *TN* triple-negative, *FFPE* formalin-fixed paraffin-embedded

According to DNA methylation array data, a total of 140 candidate markers, including 40 common BC markers, 50 luminal-dominant markers, and 50 TN-dominant markers, were selected. Figure 1 shows the distribution of meanNC and meanBC among the whole of the array probes (*n* = 482,421), and the distribution of meanLum and meanTN in the array probes with meanNC <0.05 (*n* = 121,079). The colored dots represent the selected 140 candidate markers. The methylation values of these markers are shown in a heat map format in Fig. 2. All the selected candidates had a low methylation status in the non-BC samples (ß-value <0.05), shown in blue. Some of the luminal-dominant marker candidates were highly methylated, even in the TN samples, probably because the luminal androgen-receptor-positive subtype samples may be included in these TN samples.

### Validation of candidate DNA methylation markers using the public database

As the number of samples of FFPE tissue specimens was relatively small, we validated the methylation status of the candidate markers using relatively large public datasets. There were 143 samples of PBMC in GSE 58888 [23] and 610 samples of BC in TCGA [24] datasets using the Illumina Infinium Human Methylation 450 BeadChip Assay platform. In 610 samples of TCGA data, we analyzed 213 samples which were linked with subtype information of PAM50. In the BC samples, the numbers of samples in the luminal A, luminal B, HER2-enriched, normal-like, and basal-like subtypes were 140, 46, 14, 5, and 40, respectively. A heat map generated using these datasets demonstrated that luminal A/B and PBMC samples had a similar methylation pattern to our luminal samples and blood samples (Additional file 3: Figure S2A). The basal-like subtype in PAM50 and the clinical TN subtype are not identical but partially overlaped; they also had a similar methylation pattern in our analysis. Although the TCGA samples were not laser-microdissected, the methylation pattern was similar, which indicated that methylation marker status would not be affected by the contaminated stromal cells.

### Screening of selected candidate markers

**Fig. 3:**
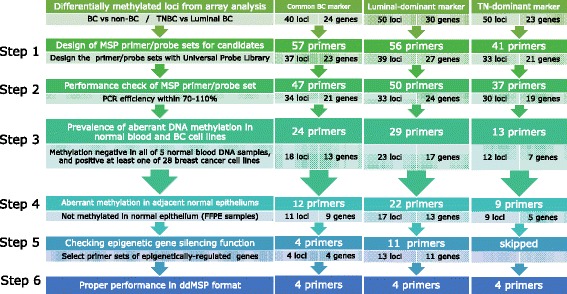
Screening of epigenetic markers. *BC* breast cancer, *TNBC* triple-negative breast cancer, *MSP* methylation-specific PCR, *FFPE* formalin-fixed paraffin-embedded, *ddMSP*, droplet digital methylation-specific PCR

The steps for screening the selected candidate markers are illustrated in Fig. 3. Briefly, the screening steps included (1) a primer/probe quality check, (2) quantitative MSP screening using BC cell lines and normal blood samples, (3) quantitative MSP screening using laser-microdissected FFPE samples of normal epithelia, (4) checking the gene silencing function of candidate markers, and (5) checking the signal amplitude pattern in ddMSP reactions. We selected *JAK3*, Ras-specific guanine nucleotide-releasing factor 1 (*RASGRF* *1*), carboxypeptidase X (*CPXM1*), and Src homology 2 domain-containing adapter protein F (*SHF*) as the common BC markers, Dynamin 3 (*DNM3*), Caveolin 2 (*CAV2*), Homeobox protein Hox-A10 (*HOXA10*), and *B3GNT5* as the luminal-dominant markers, and *ST3GAL6*, Dachshund homolog 1 (*DACH1*), P2X purinoceptor 3 (*P2RX3*), and chr8:23572595 as the TN-dominant markers (Table 2, Additional file 2: Tables S4 and S5). DNA methylation status and a differentially methylated region in the genomic area surrounding the selected markers are illustrated in Additional file 3: Figure S3. For the common BC markers and the luminal-dominant markers, we selected the markers possessing an epigenetic gene silencing function (Additional file 3: Figures S4 and S5). Selected subtype-specific methylation markers are statistically significantly differentially methylated in the luminal and basal subtypes in the TCGA dataset [24] (Additional file 3: Figure S2-B).

**Table 2:** Epigenetic markers employed in the ddMSP assay

Gene	Name	Product length	Chr	CpG
Common BC markers				
*JAK3*	Tyrosine-protein kinase JAK3	129	19	s-shore
*RASGRF1*	Ras-specific guanine nucleotide-releasing factor 1	104	15	island
*CPXM1*	Carboxypeptidase X1	94	20	island
*SHF*	Src homology 2 domain-containing adapter protein F	117	15	island
Luminal-dominant markers				
*DNM3*	Dynamin 3	105	19	n-shore
*CAV2*	Caveolin 2	141	7	island
*HOXA10*	Homeobox protein Hox-A10	135	7	island
*B3GNT5*	UDP-GlcNAc:betaGal beta-1,3-N acetylglucosaminyltransferase 5	97	3	island
TN-dominant markers				
*ST3GAL6*	ST3 beta-galactoside alpha-2,3-sialyltransferase 6	82	3	s-shore
*DACH1*	Dachshund homolog 1	110	13	n-shore
*P2RX3*	P2X purinoceptor 3	118	11	island
Chr8:23572595	Intergenic locus corresponding to probe cg23495581, located at chr 8: 23,572,595 in GRCh37	83	8	s-shore
Internal control markers for MSP				
*CREM*	cAMP-responsive element modulator	75	10	
*GLYATL3*	Glycine N-acyltransferase-like protein 3	107	6	
*ELMOD3*	ELMO/CED-12 domain containing 3	90	2	
*KLF9*	Kruppel-like factor 9	89	9	

*ddMSP* droplet digital methylation-specific PCR, *Chr* chromosome, *island* CpG island, *shore* CpG shore (region within 2000 bps from CpG island), *n-shore/s-shore* northern/southern CpG shore (CpG shore attached to upstream/downstream side of CpG island, respectively), *GRC* Genome Reference Consortium

### Performance of the internal control marker panel

In this study, we adopted an internal control marker panel to assess the concentration of cfDNA in the plasma sample. For precise assessment of cfDNA concentration, the markers should not be affected by copy number alteration (CNA) of the cancer genome. Therefore, we chose four markers (cAMP-responsive element modulator (*CREM*), Glycine N-acyltransferase-like protein 3 (*GLYATL3*), ELMO/CED-12 domain containing 3 (*ELMOD3*), and Kruppel-like factor 9 (*KLF9*)), for which the CNA rates were less than 5% in BC, according to the cBioPortal database. A geometric mean of four markers represented the cfDNA concentration of the samples. We compared the performance of this internal control panel and conventional *ACTB* primer/probe in the ddMSP assay system, using white blood cell DNA samples derived from 16 HVs and 16 patients with BC (Additional file 3: Figure S6). There was good correlation between cfDNA concentration measured by this panel and by *ACTB*. However, the amounts of cfDNA detected by the panel were significantly higher than by the *ACTB* primer/probe set.

### Development of a BC detection model using the ddMSP system

First, the detection performance of each variable was assessed by univariate analysis (Additional file 3: Figure S7A and B). The AUC for methylation markers in the ROC analysis ranged from 0.56 to 0.71. The AUC of each internal control surpassed 0.80, and the AUC for the mean of the internal controls was 0.89. The AUCs for the mean value of the 12 methylation markers and the number of positive methylated markers were 0.77 and 0.82, respectively.

**Fig. 4:**
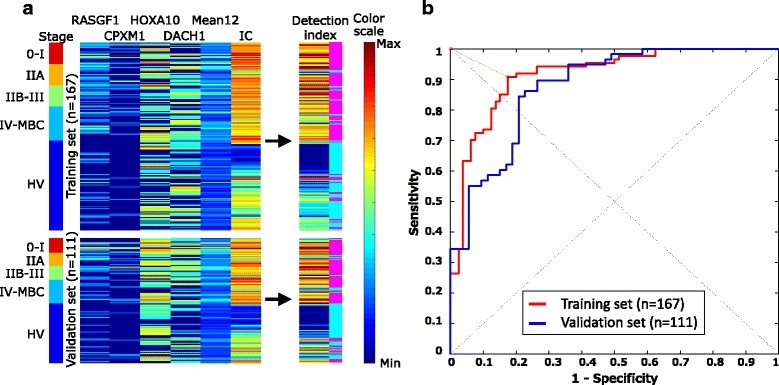
Droplet digital methylation-specific PCR (ddMSP) data and receiver operating characteristic (ROC) curves of the best support vector machine (SVM) model to distinguish patients with breast cancer (BC) from healthy volunteers (*HV*). **a** ddMSP data (*heat map* format). Cases are sorted by stage as the *ribbons* (*left*) indicate. In the *left heat map*, the *color* of each *square* represents the log10 concentration of the selected variables in the best SVM model. In the *right heat map*, the *color* of each *square* represents the detection index. Positive (cancer) and negative (non-cancer) calls from the detection index are shown in the *right side* of the detection index in *pink* and *light blue*, respectively. **b** In the ROC curve analysis, cases with a detection index of more than −0.07923 were defined as positive for BC. *Red* and *blue lines* indicate the ROC curve of the training and validation sets, respectively. *MBC* metastatic breast cancer

Optimization of variable combinations is important to obtain a detection model with high accuracy. In this study, we tested all of the possible combinations using 15 variables (n = 2^15^ − 1) by the LOOCV method. The SVM model using *RASGRF*﻿*1*, *CPXM1*, *HOXA10*, and *DACH1*, the mean of 12 markers, and the mean of the internal controls had the highest AUC of 0.92, thus, we selected this variable combination from the detection models. The sensitivity and specificity of this model was 0.91 and 0.83, respectively (Additional file 2: Table S8). The equation of the selected SVM model is expressed below:

$$ \mathrm{Detection}\mathrm{index}=0.62449\times \left[\mathrm{RASGRF}1\right]+0.78110\times \left[\mathrm{CPXM}1\right]+0.12115\times \left[\mathrm{HOXA}10\right]+0.36760\times \left[\mathrm{DACH}1\right]+0.65288\times \left[\mathrm{Mean}12\right]+2.44704\times \left[\mathrm{I}\mathrm{C}\right]-6.98073 $$

where [RASGRF1], [CPXM1], [HOXA10], [DACH1], [Mean12], and [IC] represented the log10 concentration of the methylated DNA fragments of *RASGRF1*, *CPXM1*, *HOXA10*, *DACH1* gene loci, mean concentration of 12 methylation markers, and the mean concentration of four internal control markers, respectively. According to the ROC curve analysis, samples with a detection index of more than −0.07923 were defined as positive for BC. All the ddMSP data are shown in Additional file 2: Table S7 and are also presented in a heat map format (Fig. 4a and Additional file 3: Figure S7C). The pattern of IC was similar to that of the detection index, which indicated that the IC largely contributed to the detection index. However, their patterns were not the same. Thus, other epigenetic markers might contribute to increasing specificity of the model.

As a validation study, the developed SVM model was applied to the validation dataset. The AUC, sensitivity, and specificity of the validation set were 0.88, 0.84, and 0.79, respectively. Using all the data, the sensitivity and specificity of this model was 0.88 and 0.81, respectively. In addition, the positive/negative predictive values and accuracy of the model was 0.84, 0.85, and 0.85, respectively (Additional file 2: Table S8). The ROC curves of the selected model for the training and validation sets are shown in Fig. 4b.

### Age bias in the detection index in patients with BC

The cells of elderly individuals tend to be hypermethylated, compared to the cells of younger individuals. In addition, the HVs who participated in this study were significantly younger than the patients with BC. To determine whether the detection accuracy of this model was biased by age, we tested correlation between the detection index and age in the BC samples. As shown in a scatter plot (Additional file 3: Figure S8), there was almost no relationship between age and the detection index (Pearson’s correlation, *r* = 0.075, *p* = 0.39). There was weak but significant correlation between age and 3 out of the 12 markers, and as a single marker (Additional file 2: Table S9). Among the six variables employed in the fixed model, only *RASGRF* *1﻿* was biased by age. This may be the reason why the detection index was not biased by age as a whole.

### Correlation between stage, subtype, and the detection index

**Fig. 5:**
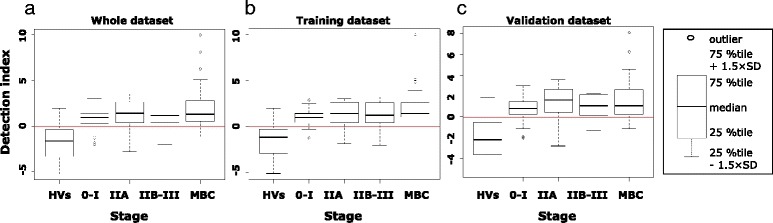
Distribution of detection indexes according to breast cancer stage. Detection indexes of patients with breast cancer were significantly higher than in healthy volunteers (*HVs*) both in the training and validation sets. Data are also shown in boxplot format (*right*). *Red line* cutoff for positive/negative diagnosis. *MBC* metabolic breast cancer

There was a statistically significant trend toward a higher detection index in advanced-stage BC samples (Jonckheere-Terpstra (JT) test, *p* = 0.0087) (Fig. 5). However, this did not mean that the samples in the early stages tended to be diagnosed as false negatives. All four patients with ductal carcinoma in situ (DCIS) and 85% of patients with stage-I BC were correctly categorized into the cancer group. For more detail, in 41 of the 47 patients with stage-I cancer, the size of the primary tumor was recorded; the sensitivity for patients with T1a (*n* = 2), T1b (*n* = 12), and T1c (*n* = 27) BC was 1, 0.83, and 0.85, respectively. Furthermore, the AUC of ROC analysis of early BC was 0.911 in the training set and 0.854 in the validation set, which was comparable with the AUC for advanced BC, ranging from 0.896 to 0.960 in the training set, and from 0.881 to 0.901 in the validation set (Additional file 3: Figure S9).

The research aim of this study was to develop a tool for the early detection of BC. Thus, the detection accuracy in these early-stage samples would have significant impact for future clinical application. As our detection index correlated with the stage of BC, the index might indicate the prognosis of patients with BC. However, all of the cfDNA samples were collected after December 2011. Thus, the follow-up period was too short to derive any statistical conclusions about survival in BC.

**Fig. 6:**
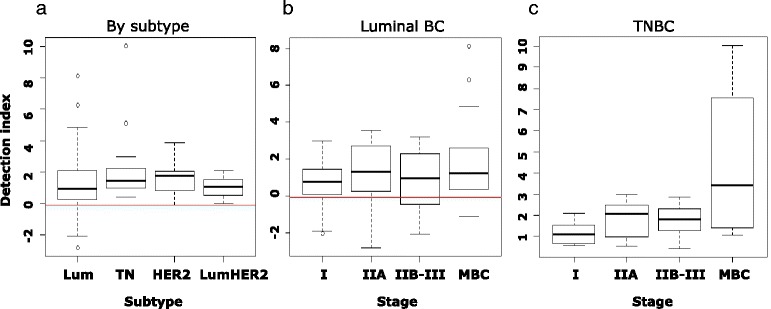
Distribution of detection indexes by subtype and stage. **a** There was no statistically significant trend in the distribution patterns of the detection indexes among the four breast cancer (BC) subtypes. **b** Distribution of detection indexes of patients with luminal BC by stage. **c** Distribution of detection indexes of patients with TN BC by stage. *Lum* luminal, *TN* triple-negative, *HER2* epidermal growth factor receptor 2

The detection index did not differ among the four BC subtypes (Kruscal-Wallis test, *p* = 0.074) (Fig. 6a). In the TN BC cases, there was also a significant trend toward a higher detection index in samples from patients in the advanced stage of BC (JT test, *p* = 0.021) (Fig. 6c), whereas there was no such significant trend in the patients with luminal BC (JT test, *p* = 0.05) (Fig. 6b). There was also no trend in the patients with HER2 and luminal HER2 BC (JT test, *p* = 0.20 and *p* = 0.81, respectively), which is likely due to the small sample size (Additional file 3: Figure S10).

All the TN, HER2, and luminal HER2 BC samples, even those at stage I, were stratified into the cancer group. In contrast, 17 out of the 98 luminal BC samples (17.3%) were falsely stratified into the non-cancer group, which contained some advanced/metastatic cases (Fig. 5). Limited to the early stage, 7 patients (19.4%) were diagnosed as non-cancer. In the 17 false-negative patients, 15 had >50% estrogen-receptor-positive cells, and 13 also had >20% progesterone-receptor-positive cells in primary tumors. Furthermore, the Ki-67 index of 10 false-negative patiets was under 14%. Taken together, the fixed model had relatively lower detection accuracy for the luminal A-like subtype [27] of BC samples than that for the other subtype samples.

## Discussion

In this study, we developed a cfDNA-based system for early diagnosis of BC using an epigenetic marker panel. Most previous studies of DNA methylation markers in cfDNA for BC utilized the MSP method by real-time PCR, and obtained a wide range of diagnostic accuracy, as shown in Additional file 2: Table S10 [12–15, 17–20]. Generally, methylation markers were detected frequently in patients with metastatic BC, unlike in patients with early BC, in whom methylation markers were less frequent.

In comparison with other studies, our study had unique and advantageous key points. First, we selected epigenetic markers from genome-wide screening by array analysis, whereas most previous studies chose markers in a knowledge-based way. Screening novel markers from genome-wide analysis required considerable effort to identify the final markers; however, this method may have a better chance of obtaining accurate marker sets than the knowledge-based method. Furthermore, we validated the results of our methylation array analysis, using large sample cohorts of PBMC samples [23] and TCGA BC samples [24]. This validation analysis confirmed that selected candidate markers were differentially methylated among subtypes in independent datasets.

Second, we employed the cfDNA concentration data in the detection algorithm to improve detection performance. The mean IC, which represented the cfDNA concentration, largely contributed to the high accuracy of the algorithm. Third, our model was highly accurate even in the detection of patients with early BC. The sensitivity of detection in patients with BC stage 0-I was 90.0% in the training set and 81.0% in the validation set. The ROC of AUC for this stage was 0.911 in the training set, and 0.854 in the validation set. In the previous studies conducted in the USA, Europe, and Asia, the sensitivity and specificity of mammography ranged from 74.6 to 92.5% and from 83.1 to 99.5%, respectively [5, 28–30]. In addition, the sensitivity and specificity of mammography in women aged 40–49 years was lower than in women aged 50–70 years [2]. Taking into consideration that 42% of patients (*n* = 117) in this study was below 50 years of age, the detection of early BC by our model was comparable with that of mammography. Thus, these results indicated that our system could be an optional method in BC mass screening in the future. Finally, we validated the accuracy of the fixed model using a large cohort (*n* = 111). We proved that our system could have generalized potential to distinguish patients with BC from HVs.

Similar to other reports, each methylation marker in this study had low-range to mid-range sensitivity. The low sensitivity is reasonable because we intentionally selected luminal-dominant and TN-dominant markers that were unmethylated in the other subtypes. In general, the keys to building a good multi-marker mathematical model for prediction or diagnosis include avoiding over-fitting to obtain a generalized model, and covering as large a variety of data patterns as possible. According to the results of the TCGA Project [24] and the Carolina Breast Cancer Study [31], there are some subtypes within the DNA methylation pattern. If we chose markers only by sensitivity as a single marker, epigenetic data from these markers would be redundant and would miss some important features. Thus, we intentionally selected subtype-specific markers, not only common BC markers. Adding different types of information, such as mean methylation values and a cfDNA concentration measured by internal control markers, helped to improve the accuracy of the model. Moreover, the numbers of variables are important. The model should include enough data to accurately show the potential variety without overfitting the model. Sixteen markers, the number used in our model, would be a reasonable size, and feasible in terms of clinical application by the PCR-based assay system, similar to Oncotype Dx [32].

Markers targeting four genes were employed in the fixed model. Three of the four genes were recognized as tumor suppressor genes according to previous functional studies. RASGRF1 activates Ras by stimulating the dissociation of GDP from RAS protein. RASGRF1/2 regulates Cdc42-mediated tumor cell transformation and cell motility, working as a tumor suppressor gene [33]. Hypermethylation in the promoter region of *RASGRF1* has been observed in gastric cancer cells and precancerous tissues of the gastric mucosae [34]. Our report is the first to show that the *RASGRF1* promoter region is hypermethylated in both the luminal and TN BC subtypes.

*CPXM1*, also known as *CPX1*, encodes a metallocarboxypeptidase protein. Although one study reported that CPXM1 may regulate osteoclastogenesis in mice [35], its function in human cancer cells remains unknown. Our analysis indicates that its expression is epigenetically regulated, and it may act as a tumor suppressor gene in BC cells. However, further functional studies are required to confirm its function.

*HOXA10* encodes one of the DNA-binding transcription factors that regulate gene expression, morphogenesis and differentiation, functioning as a tumor suppressor gene. *HOXA10* is methylated in differentiated CD24-positive normal mammary cells and luminal BC cells [36], and the methylation level increases during the progression of BC from DCIS via a primary invasive ductal carcinoma, to a metastatic tumor [36, 37]. These data are consistent with our results, that *HOXA10* is a luminal-dominant marker.

*DACH1* encodes a chromatin-associated protein that regulates gene expression and cell fate determination during development, and also functions as a tumor suppressor gene. *DACH1* is epigenetically silenced in colorectal and hepatocellular carcinoma [38, 39]. In BC, DACH1 represses aggressive characteristics such as stem cell function, epithelial-mesenchymal transition, migration activity, and so on [40–45]. Moreover, DACH1 expression is higher in the luminal subtype than in the basal subtype [42, 43, 46]. These facts support our observation that *DACH1* is selected as a TN-dominant methylation marker.

This panel also contained four novel internal control markers for MSP to measure cfDNA concentration precisely. The primer/probe sets were designed to target DNA sequences with no CpGs. In this study, the mean value of these internal controls had a good AUC, which largely contributed to the high detection accuracy of the developed SVM model. This finding was consistent with previous articles showing that the cfDNA concentration in patients with BC was significantly higher than that of HVs [47–50]. However, the methods in these previous results have not been implemented in BC screening. As the quantity of DNA was measured by spectrophotometry or PCR in these studies, the data may not have been accurate enough to detect early BC. In the present study, we employed a digital PCR system to enable absolute quantification of the amount of cfDNA and aberrantly methylated DNA fragments. The mean of the internal controls had a high AUC as a single marker, contributing to the development of a more accurate algorithm by adding information to cfDNA methylation data. According to the cBioPortal data, the genes of the internal control markers were mutated, amplified, and lost in less than 5% of other malignancies [25, 26]. Thus, this internal control panel could be beneficial for the detection of other types of cancer as well.

On the other hand, this ddMSP-based detection system has some limitations. First, there were 23 (15.5%) false positives among the HVs. Although methylation markers were selected with an emphasis on specificity, some methylation markers have low specificity. One explanation is the non-specific elevation of cfDNA concentration. In fact, the cfDNA concentration in the false-positive HVs was significantly higher than the true-negative HVs (Additional file 3: Figure S11). According to the coefficients of the model equation, the contribution of cfDNA concentration to the detection index is large. Thus, elevated cfDNA concentration caused by non-cancerous events such as inflammation or a benign cell-proliferative lesion may result in a false-positive diagnosis. Another possible reason is the existence of a pre-diagnostic malignant lesion, and not only BC. Our clinical data contained the BC screening results of the HVs by imaging and physical examination, which could not deny the existence of pre-diagnostic BC or other malignancies. Longitudinal analysis using serially obtained samples is required to check whether false-positive individuals have such lesions. However, the false-positive rate in this study was within a comparable level to current BC screening methods based on clinical breast examination and imaging, such as mammography and ultrasonography, with specificity ranging from 6.9 to 19.6% [2, 3, 5].

Second, there were 17 (17.3%) false-negative patients with luminal BC, which included some with advanced/metastatic BC, and 7 (19.4%) were limited to early BC. The false-negative patients had low mean values of 12 methylated markers (*t* test, *p* < 0.0001) and low cfDNA concentration (*t* test, *p* < 0.0001) (Additional file 3: Figure S11). Ten false-negative patients were categorized as having the luminal A-like subtype of BC. Due to the fact that in patients with cancer, cfDNA may consist of circulating tumor DNA derived from the necrotic or apoptotic tumor cells and cell-free DNA from cells in the tumor microenvironment, luminal BC with low proliferation and low activity in its tumor microenvironment might produce relatively low cfDNA, and may cause false-negative diagnosis.

Pepe, et al., statisticians in the Early Detection Research Network (EDRN), defined five phases of screening biomarker development, and described the aims, study design, and evaluation methods for each phase. According to these definitions, this study was in phase 1 (preclinical exploratory studies) and phase 2 (clinical assay development for clinical disease) [51]. The usefulness of this system in the BC screening setting should be demonstrated in the later phases. According to our results, this detection system for BC seems to be worthwhile for advancement into the next phase.

The original objective of this system was early detection of BC for screening purposes. However, this system can be applied to clinical uses other than for detection of BC. Previous DNA methylation studies using cfDNA demonstrated that methylation status of several genes was different at baseline in responders and non-responders to therapy, and the methylated DNA marker decreased in responders during therapy [16]. In the present study, as cfDNA samples in the more advanced stages had a higher detection index, the index represented tumor burden. Thus, this ddMSP system could also be a useful tool to monitor the therapeutic response of metastatic BC. Furthermore, this panel could distinguish early TN BC, and could have potential as an alternative to screening by magnetic resonance imaging in patients and carriers of the *BRCA*-mutation. These issues will be investigated in a further study.

## Conclusion

We established an epigenetic marker panel for cfDNA and a detection algorithm to distinguish patients with BC from HVs with high accuracy. As the detection of early BC using this system was comparable with mammography screening, this cfDNA-based detection system would be beneficial as an option for BC screening. A further study is necessary to demonstrate its clinical usefulness as an optional method for BC screening.

## Additional files

Additional file 1: This file provides detailed materials and methods for this study. (DOCX 66 kb)
Additional file 2: Table S1. Characteristics of cell lines used in this study. **Table S2.** Characteristics of FFPE samples used in methylation array analysis. **Table S3.** Characteristics of healthy volunteers in methylation array analysis. **Table S4.** Primer/probe set designed for methylation markers. **Table S5.** Sequence of primer/probe sets for methylation markers. **Table S6.** Sequence of primer/probe sets of internal control markers. **Table S7.** Clinical data of cfDNA samples and results of ddMSP analysis. **Table S8.** Sensitivity and specificity of the best SVM model. **Table S9.** Correlation between age and each marker/parameter. **Table S10.** Summary of recent epigenetic studies regarding cfDNA for BC. **Table S11.** Upper and lower cutoff threshold of positive droplets. **Table S12.** Cutoffs of marker concentration to categorize into marker-positive/negative groups. **Table S13.** *P* values of statistical tests in the validation analysis using public datasets. (XLSX 172 kb)
Additional file 3: Figure S1. Laser microdissection of pan-cytokeratin (AE1/AE3)-immunostained FFPE specimens. **Figure S2.** A-B Validation analysis using large public datasets. **Figure S3.** A-L DNA methylation status in genomic region surrounding candidate marker loci, and differentially methylated region. **Figure S4.** Unmasking of epigenetically silenced genes by demethylating agent and histone deacethylase inhibitor (common BC markers). **Figure S5.** Unmasking of epigenetically silenced genes by demethylating agent and histone deacethylase inhibitor (luminal-dominant markers). **Figure S6.** Relationship between *ACTB* and the new panel of internal control markers. **Figure S7.** A-C ROC curves and ddMSP data of 12 methylation markers and three parameters. **Figure S8.** Detection index and age. **Figure S9.** ROC curves in each stage of BC. **Figure S10.** Distribution of detection indexes of HER-positive patients by stage. **Figure S11.** cfDNA concentration in HVs and patients with BC. **Figure S12.** A-E Determining upper and lower thresholds for positive droplets. (PPTX 12394 kb)

## Abbreviations

5’-Aza-dC: 5’-Aza-2-deoxycytidine
AUC: area under the curve
BC: breast cancer
BORN: Breast Oncology Research Network
cfDNA: circulating cell-free DNA
CNA: copy number alteration
*CPXM1*: carboxypeptidase X (M14 family) member1
*CREM*: cAMP-responsive element modulator
CTC: circulating tumor cell
*DACH1*: Dachshund family transcription factor 1
DCIS: ductal carcinoma in situ
ddMSP: droplet digital methylation-specific PCR
*ELMOD3*: ELMO/CED-12 domain containing 3
FFPE: formalin-fixed paraffin-embedded
*GLYATL3*: glycine N-acyltransferase-like protein 3
HER2: human epidermal growth factor receptor 2
*HOXA10*: homeobox A10
HV: healthy volunteer
JT test: Jonckheere-Terpstra test
*KLF9*: Kruppel-like factor 9
LMD: laser microdissection
LOOCV: leave-one-out cross-validation
MBC: metastatic breast cancer
meanBC: mean β-values in all breast cancer samples
meanLum: mean β-values in luminal-type BC samples
meanNC: mean β-values of the non-breast cancer samples
meanTN: mean β-values in the triple-negative breast cancer samples
MSP: methylation-specific PCR
PBMC: peripheral blood mononuclear cells
PBS: phosphate-buffered saline
ROC: receiver operating characteristic
RT-PCR: reverse transcription PCR
SVM: support vector machine
TCGA: The Cancer Genome Atlas
TN: triple negative
UPL: Universal Probe Library
