# Supplementary material for: Circulating cell-free DNA-based epigenetic assay can detect early breast cancer
Source: Breast Cancer Res. 2016 Dec 19;18:129. doi: 10.1186/s13058-016-0788-z (PMC5168705; doi:10.1186/s13058-016-0788-z)
Supplement: Additional file 1: — This file provides detailed materials and methods for this study. (DOCX 66 kb) [file 13058_2016_788_MOESM1_ESM.docx]

Supplemental Information

Circulating cell-free DNA-based epigenetic assay can detect early breast cancer

Natsue Uehiro^1^, Fumiaki Sato^1,5^, Fengling Pu^2^, Sunao Tanaka^1^, Masahiro Kawashima^1^, Kosuke Kawaguchi^1^, Masahiro Sugimoto^3^, Shigehira Saji^4^, Masakazu Toi^1^

1: Department of Breast Surgery, Graduate School of Medicine, Kyoto University, Kyoto, Japan.

2: Department of Target Therapy Oncology, Graduate School of Medicine, Kyoto University, Kyoto, Japan.

3: Institute for Advanced Bioscience, Keio University, Tsuruoka, Yamagata, Japan.

4: Department of Medical Oncology, Fukushima Medical University, Fukushima, Japan.

5: corresponding author

**Detailed information of materials and methods.**

**Collection of clinical samples**

All blood and tissue samples were provided from a multi-institutional biobank project; Breast Oncology Research Network (BORN)-Biobank that was initiated and maintained by the Department of Breast Surgery, Kyoto University. Blood samples of BC patients were obtained after they received a traditional BC diagnosis. In this study, Stage 0-I BC was considered as early BC.

**Subtyping of breast cancer (BC)**

Subtypes of BC samples/patients were determined by immunohistochemistry of estrogen receptor (ER), progesterone receptor (PR), and HER2. The cutoff of ER and PR positivity was 1% each [1], and HER2 was 3+. If HER2 was 2+, FISH/DISH Her2/Cep17 ratio > 2.2 was determined as positive [2]. The Luminal subtype was defined as ER and/or PR positive and HER2 negative. The Luminal HER2 subtype was defined as ER and/or PR positive and HER2 positive. The HER2 subtype was defined as ER and PR negative and HER2 positive. The TN subtype was negative for ER, PR and HER2 [3, 4].

**Laser capture microdissection (LMD) of FFPE samples**

Individual 10-µm thick formalin-fixed paraffin embedded (FFPE) specimens of surgically resected BC tissues were placed on Leica’s foil membrane slides, and immunohistochemically stained by pan-cytokeratin antibody cocktails (AE1/AE3, Dako, Glostrup, Denmark, M3515). Histo/Zyme (Diagnostic BioSystems, Pleasanton, CA, USA, DBS-K046-15) was used for antigen retrieval. Then, 3% H_2_O_2_ in methanol was applied to specimens for elimination of endogenous peroxidase activity. A mixture of normal goat serum and bovine serum was used for blocking non-specific binding of antibodies. Monoclonal Mouse Anti-Human Cytokeratin Clones AE1/AE3 (Dako, M3515) and biotinylated Horse Anti-Mouse IgG Antibody were used as primary and secondary antibodies, respectively. A VECTOR Red Alkaline Phosphatase Substrate Kit (VECTOR Laboratories, Burlingame, CA, USA, SK-5100) was used for visualization, according to the manufacturer’s instructions.

LMD of the stained FFPE slides was performed using LMD7000 systems (Leica microsystems, Wetzlar, Gemany). As for the BC samples, cancer cell clusters were selectively microdissected (Supplemental Figure 1). For the normal samples, adjacent normal mammary epithelia and intraductal papilloma epithelia were microdissected. Adjacent normal epithelia from 10 patients were pooled as a single sample.

**Blood collection**

After enrollment, a blood sample was collected into an EDTA-2Na tube, and processed within one hour after the blood sampling. The blood sample was centrifuged at 3000 rpm for 10 min. The plasma sample was obtained from the supernatant of the blood sample, dispensed in 500 µl aliquots, then stored at −80°C. The rest of the blood containing white blood cells was processed to isolate the genomic DNA.

**DNA/RNA isolation**

Depending upon the sample types, we used different DNA/RNA isolation kits. Genomic DNA of cultured cell lines and plasma-removed blood were isolated using the PureLink Genomic DNA Mini kit (Life Technologies, Carlsbad, CA, USA) and QIAmp DNA Blood Maxi Kit (QIAGEN, Venlo, Netherlands) following the manufacturer’s instructions, respectively.

Genomic DNA of microdissected FFPE samples was isolated using NucleoSpin FFPE DNA Kit (Macherey Nagel GmbH, Düren, Germany). We modified the sample lysis step described in the manufacturer’s instructions. After adding 10 µl of proteinase K solution to the lysis buffer, we incubated the samples at 37°C for three hours, then added 10 µl of Proteinase K solution again and incubate for additional three hours as a boost. After lysing samples, we followed the manufacturer’s instructions to obtain DNA. The obtained DNA was qualified by Infinium HD FFEP DNA sample QC Kit (Illumina, San Diego, CA, USA).

The extraction of cfDNA from plasma was conducted using QIAmp Circulating Nucleic Acid Kit (QIAGEN) by a modified protocol from the manufacturer’s protocol to improve cfDNA yield. Briefly, 900 µl of thawed plasma was mixed with 100 µl of PBS, 800 µl of Buffer ACL (lysis buffer), and 100 µl of proteinase K solution, then incubated at 48°C for 18 hours with shaking. The sample was then mixed with an additional 100 µl of proteinase K solution by pulse-vortexing for 30 sec, and incubated for a further six hours. Finally, approximately 20 µl of cfDNA solution was obtained.

Genomic DNA was quantified using a NanoDrop (Thermo Scientific, Waltham, MA, USA). The cfDNA quantity was not estimated by spectrophotometry because of the low concentration. We estimated the quantity with the amount of internal controls by the droplet digital methylation-specific PCR (ddMSP) instead.

Total RNA was extracted using Trizol Reagent (Invitrtogen, Waltham, MA, USA) and PureLink® RNA Micro Kit (Invitrogen). RNA was quantified using a NanoDrop and stored at –80°C until use.

**Bisulfite Conversion of DNA**

Depending on the epigenetic assays, we used different bisulfite conversion kits and protocols.

For Illumina Infinium® Assay, bisulfite conversion of 1 µg of DNA from cell lines or blood and 300 ng of DNA from FFPE samples was performed using EZ DNA Methylation™ Kit (ZYMO RESEARCH, Irvine, CA, USA) as per the manufacturer’s instruction. In the protocol, we used an alternative incubation condition optimized for Illumina Infinium® Assay.

For screening of the MSP primers, 500 ng of DNA from cell lines was used for bisulfite conversion using an EZ DNA Methylation-Gold™ Kit (ZYMO RESEARCH). DNA from healthy volunteers (HVs) was used for bisulfite conversion manually, because large quantity of DNA was handled at once. Specifically, 20 µg of DNA was denatured in 200 µL of 0.3 M NaOH solution for 15 min at 37°C. The denatured DNA was added into the mixture of 1070 µl of 4 M sodium bisulfite, 70 µl of 10 mM hydroquinone, and 7.2 µl of 5 M NaOH, then incubated at 64°C for 16 hours. After incubation, 6 ml of M-Binding Buffer (ZYMO RESEARCH) and DNA sample were mixed and filtrated with a DNA-binding column (ZYMO RESEARCH DNA Clean and Concentrator-25). The column was washed with 80% Ethanol by vacuum processing, then incubated with 0.1 M NaOH diluted by 90% Ethanol for 15 min at room temperature. The column was washed with 80% Ethanol twice by vacuum processing and centrifuged at 18000×g for one min. DNA was eluted by 70 µl of Nuclease Free Water (NFW) (QIAGEN) by centrifugation at 12000×g for one min. DNA was quantified using a NanoDrop and stored at −20°C until use.

For ddMSP, all of the eluted plasma DNA samples were used for bisulfite conversion. Bisulfite conversion was done using an EZ DNA Methylation-Gold™ Kit as per manufacturer’s instruction. DNA was eluted with 22 µl of NFW. Bisulfite converted DNA was stored at −20°C until use.

**Comprehensive DNA methylation profiling**

We conducted an Illumina Infinium Human Methylation 450 BeadChip Assay (Illumina), following the manufacturer’s protocols optimized for sample types, such as the Infinium HD Assay Methylation Protocol for cell lines and blood samples, and the Infinium HD FFPE Restore Protocol and Infinium HD FFPE Methylation Assay Protocol for FFPE samples. Then, we obtained comprehensive DNA methylation profiling of 56 laser-microdissected FFPE samples (38 Luminal, four Luminal HER2, one HER2, 11 Triple negative [TN] -type BCs, one pooled normal epithelia, and one intraductal papilloma), 34 samples of DNA from 31 cultured cells (four Luminal, three Luminal HER2, two HER2, 18 TN, one unknown type BC, and three non-BC cells ) and 29 white blood cell DNA samples from HVs, as listed in Supplemental Tables 1-3. The peak bias in β-values of the two different probe types was corrected by an NIMBL toolbox for MATLAB software [5].

At the selection of candidate markers, we attached importance to the difference of the methylation patterns based on the BC subtypes. To build a generalized multi-marker mathematical model for diagnosis and avoid an over-fitting phenomenon, it is important to use several types of variables. Thus, we decided to select candidate markers from subtype-specific methylation loci, not only from commonly methylated loci in BC.

The mean β-values of the non-BC samples (meanNC), whole BC samples (meanBC), as well as the Luminal-type (meanLum), and TN-type (meanTN) of the BC samples, were calculated. We selected the candidate markers from array probes with meanNC < 0.05. The additional selection conditions of candidate markers were as follows; (a) top 20 loci of the widest gap between meanBC and meanNC; (b) top 20 loci of the lowest meanNC with meanBC > 0.6; (c) top 50 loci with largest values of meanLum – meanTN; and (d) top 50 loci with largest values of meanTN – meanLum. We referred to (a) and (b) as common BC markers, (c) as Luminal-dominant markers, and (d) as TN-dominant markers. Since the proportions of the cell lines and FFPE samples were different in the Luminal and TN samples, direct calculation of mean by sample type would be biased. To avoid such a bias, the mean β-values of each group were calculated as an average of the mean of the cell line samples and the mean of the FFPE samples. To evaluate the statistical significance of these markers, we calculated the p-values by Welch’s t-test (Supplemental Table 4).

The data discussed in this publication have been deposited in NCBI's Gene Expression Omnibus [6] and are accessible through GEO Series accession number GSE87177 (https://www.ncbi.nlm.nih.gov/geo/query/acc.cgi?acc=GSE87177).

**Screening process of DNA methylation markers**

The whole screening process of DNA methylation markers is illustrated in Figure 3.

**Step 1.** In this step, for 140 candidate loci selected in the methylation array analysis, primer/probe sets of MSP were designed. We used a Taqman-based MSP method, because MSP reactions easily produce non-specific amplifications that can be detected using the SYBR green method. To save screening costs and time, we utilized the Universal Probe Library (UPL, Roche Diagnostics GmbH, Mannheim, Germany) to design Taqman-based MSP primers and probe. As the sequence variety of UPLs is limited, we designed primers and probes as close as possible to the candidate loci selected by the methylation array analysis (Supplemental Table 4).

**Step 2.** In this step, we tested functional quality of the designed primer/probe sets for quantitative MSP reactions. The MSP reaction mix consisted of 10 µl of FastStart Universal Probe Master (ROX) (Roche Diagnostics GmbH), 1 µl of primer mix for MSP (finally 0.5 µM), 0.4 µl of UPL probe, 2 µl of template bisulfite treated DNA, and H_2_0 up to 20 µl in total. The PCR reaction was performed using the StepOnePlus Real-Time PCR System (Applied Biosystems, Foster City, CA, USA) as follows; one cycle at 95°C for 10 min, fifty cycles of 95°C for 15 sec and 60°C for 1 min. A standard curve was generated using serially-diluted fully-methylated DNA synthesized by SssI methyltransferase (New England Biolabs, Ipswich, MA, USA), and methylation values were normalized by MSP values of ACTB, as previously described [7]. Primer/probe sets within a range of PCR efficiency, 70-110%, were advanced to the next step.

**Step 3.** In this step, we analyzed the prevalence of aberrant DNA methylation in candidate loci. Using five normal white blood cell DNA samples, one primary cultured cell strain of normal breast epithelial cells (HMEpiC, ScienCell Research Laboratories, Carlsbad, CA, USA), two normal endothelial cell strains (HUVEC and HMMEC [Human Mammary Microvascular Endothelial Cells] from ScienCell Research Laboratories), and 28 BC cell lines, quantitative MSP analysis was carried out for each candidate selected in step 2. Candidate loci that were methylated in one or none of the five blood DNA samples and normal cell strains, and that were methylated in more than one BC cell lines, were selected for the further screening steps.

**Step 4.** In this step, we also checked whether candidate loci were unmethylated in normal breast epithelia, or not. We used one pooled DNA sample obtained by laser microdissection from 10 FFPE specimens of adjacent normal breast epithelia of BC patients. This pooled DNA sample was the same one analyzed in the methylation array analysis. Candidate loci that were not methylated in normal breast epithelia were chosen for the next step.

**Step 5.** In this step, we checked whether the epigenetic status at candidate loci regulated the gene expression, utilizing a pharmacologic unmasking method. On day 0, 0.5 × 10^5^ cells of MDA-MB-231 and 1 × 10^5^ cells of MCF7, T47D, and Hs578T cells were seeded in a 6-well format. On days 1–2, cells were treated with the demethylating agent 5’-Aza-2-deoxycytidine (5’-Aza-dC) (Sigma-Aldrich, St. Louis, MO, USA) at 1 µM for 48 hours. On day 3, cells were treated with both 5’-Aza-dC and histone deacetylase inhibitor trichostatin A (Sigma-Aldrich) at 300 nM for 24 hours. DNA and RNA samples were then extracted. The methylation status of each selected marker was measured by quantitative MSP described above.

Primer/probe sets for RT-PCR were designed so that UPL could be used as TaqMan probes, and that forward and reverse primer sites were located on different exons of target genes. The reaction mix was set up as follows: 10 µl of QuantiTect Probe RT-PCR Master Mix (QIAGEN), 0.8 µl of primer mix for RT-PCR (final concentration: 0.4 µM), 0.4µl of UPL probe, 0.2 µl of RT-Mix (QIAGEN), 2 or 4 µl of template RNA, and NFW up to 20 µl in total. The PCR reaction was performed using the StepOnePlus Real-Time PCR System as follows; one cycle at 95°C for 15 min, 45 cycles of 95°C for 15 sec and 60°C for 1 min. A standard curve was generated using serially diluted RNA samples of BC cell lines that highly expressed genes of interest. The expression data was normalized using a commercially available primer/probe set for 18S rRNA (Applied Biosystems) used as an internal control. Candidate loci of which methylation status and gene expression were inversely correlated were selected. For TNBC markers, because we could not find good positive control cells, this step was skipped.

**Step 6.** In this step, we checked amplification performance in ddMSP format. For the remaining candidates, we compared several features of droplet amplitude patterns by singleplex ddMSP reaction, such as separation between positive and negative droplets, amount of droplets with non-specific amplification in no-template control reaction, quantifiable range assessed by serially-diluted fully-methylated samples, and more. Conditions for ddMSP reaction are described below.

Finally, by holistic judgment of these droplet patterns, we fixed four loci for each common BC, Luminal-dominant, and TN-dominant marker.

**Validation of candidate DNA methylation markers with public data base**

To evaluate the universality of the candidate markers, we analyzed the methylation data of peripheral blood mononuclear cells (PBMCs) generated by Marttila et al. [8] (data accessible at NCBI GEO database, accession GSE58888), and that of breast cancer in The Cancer Genome Atlas (TCGA) Project [9] generated by the TCGA Research Network: <http://cancergenome.nih.gov/>. The level 3 Data Archives of HumanMethylation450 were downloaded from <https://tcga-data.nci.nih.gov/docs/publications/brca_2012/> (The data freeze November 11, 2011) as the TCGA methylation data.

We showed the methylation pattern of samples with candidate markers in a heat map format. As for the selected methylation markers, the distributions of the β-values were compared among PBMC samples, all cancer samples, Luminal BC samples, and Basal-like BC samples by Welch’s t-test.

**Selecting internal control markers**

In epigenetic research, a primer/probe set, which was developed by Eads et al. and targets the upstream region of ACTB [7] was traditionally used as an internal control reaction of MSP and also used in marker screening steps. However, in the present study, the amplification efficiency of this primer/probe set was not sufficient. In addition, because the amounts of loaded cfDNA samples in ddMSP reactions is unknown and considerably varied, precise quantification is very important in a detecting assay system. Therefore, we developed a panel of four novel internal control markers. We selected four internal control genes of which copy number alteration ratios less than 5%, according to cBioPortal database (http://cbioportal.org) [10, 11]. The primer/probe sets for internal control markers were designed to target no-CpG-containing genomic regions in order to amplify the region regardless of methylation status (Supplemental Table 6).

**ddMSP protocols**

For a simplex ddMSP, the reaction mix was set up as follows: 11 µl of ddPCR™ Supermix for Probes (Bio-Rad, Hercules, CA, USA) and 1.1 µl of forward and reverse primer mix for MSP (final concentration: 0.5 µM), 0.44 µl of TaqMan probe, 2.2 µl of bisulfite-converted cfDNA from plasma, and NFW up to 22 µl in total. For a duplex ddMSP, the amounts used of each primer and each TaqMan probe were the same as the singleplex ddMSP, and the amount of NFW was reduced. The reaction mixture and 70 µl of Droplet Generation Oil for Probes (Bio-Rad) were pipetted into the Droplet Generator DG8 Cartridge (Bio-Rad) separately. Since one cartridge had eight wells, we could evaluate 16 markers from one patient at one time. Droplets were generated by QX100 Droplet Generator (Bio-Rad). The droplet emulsion was loaded to a PCR plate, and PCR reaction was performed with a T100™ Thermal Cycler (Bio-Rad). The thermal cycling program for ddMSP was as follows: one cycle at 95°C for 10 min, fifty cycles at 94°C for 30 sec and 60°C for 1 min, one cycle at 98°C for 10 min, then stored at 4°C. After amplification, the droplets were scanned by the QX100™ Droplet Reader (Bio-Rad). The reading setting for FAM/HEX detection was selected. In every plate, reactions of SssI treated, fully methylated DNA and NFW were added as positive and negative controls, respectively.

**Establishing MSP assay using droplet digital PCR machine**

To quantify tiny amount of methylated DNA in cfDNA, we employed droplet digital PCR. To adjust selected primer/probe sets to duplex droplet digital PCR format, custom dual-labeled locked nucleic acid probes with FAM or Alexa Fluor® 532 dye and Black hole-1 quencher were synthesized for certain markers (Gene Design Inc., Ibaraki, Osaka, Japan). The final sequences of MSP primers and probes for selected markers are listed in Supplemental Table 5. The final form of the ddMSP assay consisted of eight duplex ddMSP reactions for the selected 16 markers.

**Developing a detecting algorithm using ddMSP data.**

Totally, 278 cfDNA samples from 133 HVs and 145 BC patients were enrolled and analyzed by the ddMSP assay system, and all raw droplet signal data were exported from the built-in software, and manually analyzed using MATLAB software as below. Clinicopathological characteristics of cases for cfDNA were shown in Table 1 and Supplemental Table 7.

**Step 1. Data separation into training and validation set.**
A sample data set was randomly divided into a training set (n = 167) and a validation set (n = 111), with each set being in accordance with the proportion of cancer patients and HVs, as well as BC stage. A detecting algorithm was developed using the training data set only.

**Step 2. Optimization of lower and upper thresholds for droplet amplitudes.**

In order to quantify the amount of template DNA fragments precisely, optimizing the lower and upper thresholds for droplet amplitude was important because even negative droplets have a weak fluorescent signal. Using the ksdensity function in the Bioinformatics toolbox of MATLAB software, the distribution of these negative droplets was delineated, and the peak of the negative droplets and upper limit of the peak’s range were identified for each reaction (Supplemental Figure 12-A). The signal intensity of all droplets was calculated by subtracting the upper limit value of the peak range as a baseline of droplet signals.

Scatter plots of droplet signals showed defective droplets with extremely high signal intensity infrequently (Supplemental Figure 12-B). Calculating marker concentrations with these defective droplets may lead to false positive results. Thus, to eliminate these defective droplets with an extremely high signal, we determined the upper threshold for positive droplets. In all ddMSP plates, positive control reactions using Sss-I treated, fully-methylated DNA. First, the baseline-subtracted data of all positive control reactions were concatenated into one dataset. Similarly when determining signal baseline using the ksdensity function, the distribution of these positive droplets was delineated, and the upper range limit of the positive droplet peak was defined as the upper threshold of the positive droplets (Supplemental Figure 12-C).

In the first cycle, a tentative lower threshold of positive droplets was placed at the signal baseline. The marker concentration of each reaction was then calculated with Poisson correction as previously described [12], and expressed as allele copy number of markers in 1 mL of plasma sample. According to marker concentration data of all samples in the training set, the AUC of the ROC was calculated and recorded. In the next cycle, a tentative lower threshold was raised by 10, and the AUC was calculated and recorded. These calculation cycles were repeated until the tentative lower threshold reached the upper threshold (Supplemental Figure 12-D). Among these tentative thresholds, the one with the highest AUC was defined as the optimized lower threshold of the positive droplets (Supplemental Figure 12-E). The upper and lower thresholds of the positive droplets are listed in Supplemental Table 11.

**Step 3. Determining cutoffs of marker concentration to dichotomize into marker-positive/negative groups.**

For all 12 markers, the ROC was generated using the thresholds determined in Step 2. The nearest points on the ROC from point (0, 1) were identified, and cutoffs corresponding to these points on the ROC were adopted to categorize samples into positive/negative groups. Then, for each sample, the number of positive markers was counted, and used as a parameter for detecting model building. The cutoffs for the 12 markers are listed in Supplemental Table 12.

**Step 4. Optimizing variable sets for detecting model**

All marker concentration values were converted into log10 values. Thus, the whole training data set consisted of a total of 15 variables, including the concentration values of 12 DNA methylation markers and their mean value, a mean of four internal control markers, and the number of methylation-positive markers.

We developed a detecting algorithm using a support vector machine (SVM) with a linear kernel function to distinguish cancer patients from HVs. To determine the best variable set for the model, we tested all of the variable combinations (n = 2^15^ - 1). For each combination, the detecting accuracy was estimated by leave-one-out crossvalidation (LOOCV). The model that achieved the best AUC and coefficients of each variable was > 0, was then selected as the detecting model.

We also used ordinary linear discriminant analysis (LDA) to develop the detecting model. However, detecting performance of LDA was slightly worse than SVM. The best AUC of LDA method was 0.908 for the training set. Thus, we decided to choose SVM for the model.

**Step 5. Validating the fixed detecting model using an independent data set.**

To validate the robustness of the selected model, an independent data set was used. The validation data set was prepared using thresholds of droplet signals and cutoffs of marker concentration determined by the training data set. The SVM model trained using training data containing only the best variable set was applied to the validation set. The accuracy of the detecting model for the validation set was assessed using AUC. Furthermore, we also did ROC analysis and calculated AUC to evaluate the performance of the model within each stage of BC as a subgroup analysis.

**Software used in statistical analysis and model building.**

Methylation assay analysis, data processing of ddMSP data, and algorithm construction were performed using the MATLAB software. Statistical analyses, such as correlation analysis, tendency analysis, and t-statistics, among others, were performed using R software.

Legends for Supplemental Figures

Supplemental Figure 1.

Laser microdissection of pan-cytokeratin (AE1/AE3) immunostained FFPE specimens.

Cancer cells were stained red using a VECTOR Red Alkaline Phosphatase Substrate Kit. Cancer clusters were purely microdissected as shown in the right photo, using a Leica LMD7000 system.

Supplemental Figures 2 A-B

Validation analysis using large public datasets.

For validation analysis, we obtained the TCGA datasets for breast cancer tissue samples, and GSE58888 data from GEO for blood DNA samples.
A: Heat map of 140 candidate markers.

In the heat map, the color of each square represents methylation level (ß-value) by methylation array as a color scale bar in the right side indicates. The selecting conditions of candidate markers are described in the Materials and Methods section. Luminal A/B and Basal-like samples showed similar pattern as our Luminal and TN samples, respectively. HER2-enriched and Normal like subtypes showed similar methylation pattern with Luminal subtype.

B: Distribution of β-values in 12 selected methylation markers.

The distribution of β-values was compared between PBMCs and cancer (left) and between Luminal BC and Basal-like BC (right) in each selected methylation marker.

PBMC: peripheral blood mononuclear cell

*: T-test, 10^-10^<p<10^-3^, †: T-test, 10^-20^<p<10^-10^, §: T-test, 10^-30^<p<10^-20^, ¶: T-test, p<10^-30^

Supplemental Figures 3 A-L

DNA methylation status in genomic region surrounding candidate marker loci, and differentially methylated region.

The upper genomic view of each marker was obtained from UCSC genome browser (<http://genome.ucsc.edu)>. Genomic region ranging −3000 to +3000 bps from probe point was illustrated. Red line: mean β-values of BC samples. Purple line: mean β-values of non-cancer samples. Green line: mean β-values of Luminal BC samples. Blue line: mean β-values of triple negative BC samples. DMR: differentially methylated region.

Supplemental Figure 4

Unmasking of epigenetically silenced genes by demethylating agent and histone deacethylase inhibitor (common BC markers).

Two Luminal cell lines (MCF7 and T47D) and two TN cell lines (Hs578T and MDA-MB-231) were used in this experiment. Expression amount of the gene of interest was assessed as a ratio of gene to 18s rRNA.

Supplemental Figure 5

Unmasking of epigenetically silenced genes by demethylating agent and histone deacethylase inhibitor (Luminal-dominant markers).

Two Luminal cell lines (MCF7 and T47D) were used in this experiment. Expression amount of the gene of interest was assessed as a ratio of gene to 18s rRNA.

Supplemental Figure 6

Relation between ACTB and the new panel of internal control markers.

Amounts of DNA from 30 white blood cell were quantified using ACTB primer/probe set and our new internal control panel by ddMSP. The amount of detected DNA by new internal controls was significantly higher than that by ACTB. Equation: equation of fitted line for scattered data. R^2^: coefficient of Pearson’s correlation analysis.

Supplemental Figures 7 A-C

ROC curves and ddMSP data of 12 methylation markers and 3 parameters.

A: ROC curves of 12 markers and four controls. B: left, mean of 12 methylation markers; middle, cfDNA concentration by internal controls; right, number of methylation positive markers.

C: ddMSP data are shown in a heat map format. Cases are sorted by stage as the ribbons in the left side indicates. Subtypes of cases are shown on the right of the stage ribbons. The color of each square represents the log10 concentration of the markers and parameters.

Supplemental Figure 8

Detecting index and age.

There was no significant correlation between detecting index and age.

Supplemental Figure 9

ROC curves in each stage of BC.

Upper left: Stage0-I, Upper right: Stage IIA, Lower left: Stage IIB-III, Lower right: Metastatic breast cancer (MBC).

Red and blue lines indicate ROC curve of the training set and validation set, respectively.

Supplemental Figure 10

Distribution of detecting index of HER positive patients by stage.

There was no significant tendency observed among the four different stages.

Supplemental Figure 11

cfDNA concentration of HVs and BC patients.

*: p < 0.0001 by t-test and Mann-Whitney test.

Supplemental Figures 12 A-E

Determining upper and lower thresholds for positive droplets.

A: determining a baseline signal point.

B: example of defective droplets with extremely high signals.

C: determining an upper threshold.

D: optimizing a lower threshold using moving tentative thresholds.

E: Choosing lower threshold with the best AUC in training set.

Legends for Supplemental Tables

Supplemental Table 1

Characteristics of cell lines used in this study.

Supplemental Table 2

Characteristics of FFPE samples used in methylation array analysis.

Supplemental Table 3

Characteristics of healthy volunteers in methylation array analysis.

Supplemental Table 4

Primer/probe set designed for methylation markers.

Several primer/probe sets were tested for some markers. Red sequences were used for the final marker set.

GRC: Genome Reference Consortium

Supplemental Table 5

Sequence of primer/probe sets for methylation markers.

Quencher dye for custom probes was BHQ-1.

Supplemental Table 6

Sequence of primer/probe sets of internal control markers.

Supplemental Table 7

Clinical data of cfDNA samples and results of ddMSP analysis.

Supplemental Table 8

Sensitivity and specificity of the best SVM model.

Supplemental Table 9

Correlation between age and each marker/parameter.

The results of Pearson’s correlation test are listed. RASGRF1, SHF, B3GNT5, and ST3GAL6 were age-biased.

Supplemental Table 10

Summary of recent epigenetic studies regarding cfDNA for BC.

*: sensitivity and specificity data in training set were not clearly described in the articles.

**: although the cutoff point was set for > 95% specificity for each gene, specificity as 8-gene panel was not clearly described in article.

Supplemental Table 11

Upper and lower cutoff threshold of positive droplets.

Supplemental Table 12

Cutoffs of marker concentration to categorize into marker-positive/negative groups.

*: log10-transformed concentration.

Supplemental Table 13

P-values of statistical tests in the validation analysis using public datasets.

For validation analysis, we obtained the TCGA datasets for breast cancer tissue samples, and GSE58888 data from GEO for blood DNA samples. Beta-value distribution patterns of PBMC, all type of BC, Luminal and Basal-like subtypes were compared using Welch’s t-test. PBMC: peripheral blood mononuclear cells

Reference

1. Hammond MEH, Hayes DF, Dowsett M, Allred DC, Hagerty KL, Badve S, et al. American Society of Clinical Oncology/College of American Pathologists Guideline Recommendations for Immunohistochemical Testing of Estrogen and Progesterone Receptors in Breast Cancer. J Clin Oncol. 2010;28(16):2784-2795.

2. Wolff AC, Hammond ME, Schwartz JN, Hagerty KL, Allred DC, Cote RJ, et al. American Society of Clinical Oncology/College of American Pathologists guideline recommendations for human epidermal growth factor receptor 2 testing in breast cancer. J Clin Oncol. 2007;25(1):118-145.

3. Goldhirsch A, Wood WC, Coates AS, Gelber RD, Thürlimann B, Senn HJ, et al. Strategies for subtypes—dealing with the diversity of breast cancer: highlights of the St Gallen International Expert Consensus on the Primary Therapy of Early Breast Cancer 2011. Ann Oncol. 2011;22(8):1736-1747.

4. Goldhirsch A, Winer EP, Coates AS, Gelber RD, Piccart-Gebhart M, Thurlimann B, et al Personalizing the treatment of women with early breast cancer: highlights of the St Gallen International Expert Consensus on the Primary Therapy of Early Breast Cancer 2013. Ann Oncol. 2013;24(9):2206-2223.

5. Wessely F, Emes RD. Identification of DNA methylation biomarkers from Infinium arrays. Front Genet. 2012;3:161.

6. Edgar R, Domrachev M, Lash AE. Gene Expression Omnibus: NCBI gene expression and hybridization array data repository. Nucleic Acids Res. 2002;30(1):207-210.

7. Eads CA, Danenberg KD, Kawakami K, Saltz LB, Blake C, Shibata D, et al. MethyLight: a high-throughput assay to measure DNA methylation. Nucleic Acids Res. 2000;28(8):E32.

8. Marttila S, Kananen L, Hayrynen S, Jylhava J, Nevalainen T, Hervonen A, et al. Ageing-associated changes in the human DNA methylome: genomic locations and effects on gene expression. BMC Genomics. 2015;16:179.

9. Network CGA. Comprehensive molecular portraits of human breast tumours. Nature. 2012;490(7418):61-70.

10. Cerami E, Gao J, Dogrusoz U, Gross BE, Sumer SO, Aksoy BA, et al. The cBio cancer genomics portal: an open platform for exploring multidimensional cancer genomics data. Cancer Discov. 2012;2(5):401-404.

11. Gao J, Aksoy BA, Dogrusoz U, Dresdner G, Gross B, Sumer SO, et al. Integrative analysis of complex cancer genomics and clinical profiles using the cBioPortal. Sci Signal. 2013;6(269):pl1.

12. Hindson BJ, Ness KD, Masquelier DA, Belgrader P, Heredia NJ, Makarewicz AJ, et al. High-throughput droplet digital PCR system for absolute quantitation of DNA copy number. Anal Chem. 2011;83(22):8604-8610.
