# Supplementary material for: Circulating cell-free DNA-based epigenetic assay can detect early breast cancer
Source: Breast Cancer Res. 2016 Dec 19;18:129. doi: 10.1186/s13058-016-0788-z (PMC5168705; doi:10.1186/s13058-016-0788-z)
Supplement: Additional file 3: Figure S1. — Laser microdissection of pan-cytokeratin (AE1/AE3)-immunostained FFPE specimens. Figure S2. A-B Validation analysis using large public datasets. Figure S3. A-L DNA methylation status in genomic region surrounding candidate marker loci, and differentially methylated region. Figure S4. Unmasking of epigenetically silenced genes by demethylating agent and histone deacethylase inhibitor (common BC markers). Figure S5. Unmasking of epigenetically silenced genes by demethylating agent and histone deacethylase inhibitor (luminal-dominant markers). Figure S6. Relationship between ACTB and the new panel of internal control markers. Figure S7. A-C ROC curves and ddMSP data of 12 methylation markers and three parameters. Figure S8. Detection index and age. Figure S9. ROC curves in each stage of BC. Figure S10. Distribution of detection indexes of HER-positive patients by stage. Figure S11. cfDNA concentration in HVs and patients with BC. Figure S12. A-E Determining upper and lower thresholds for positive droplets. (PPTX 12394 kb) [file 13058_2016_788_MOESM3_ESM.pptx]

## Slide 1
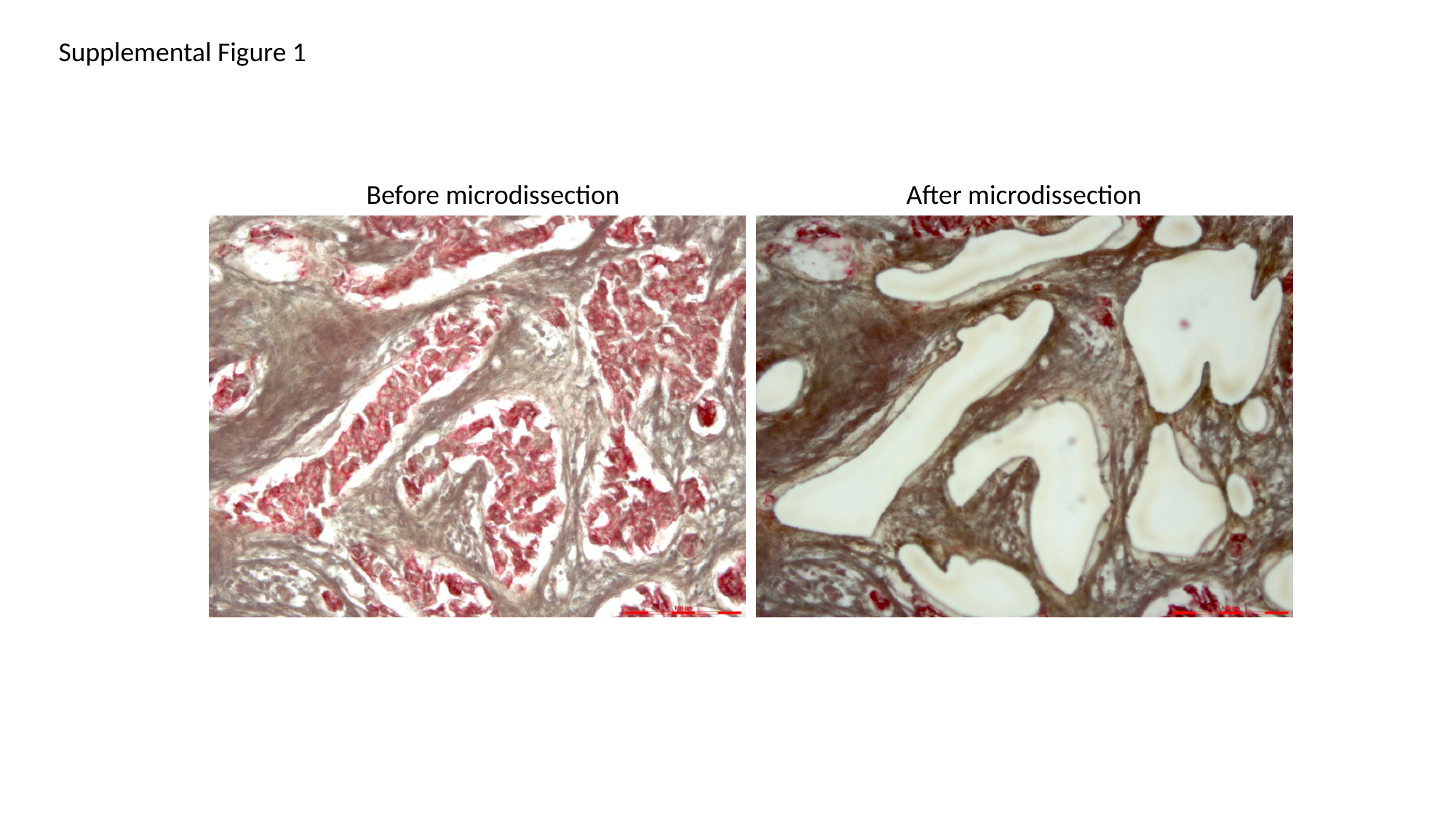

Supplemental Figure 1
Before microdissection
After microdissection

## Slide 2
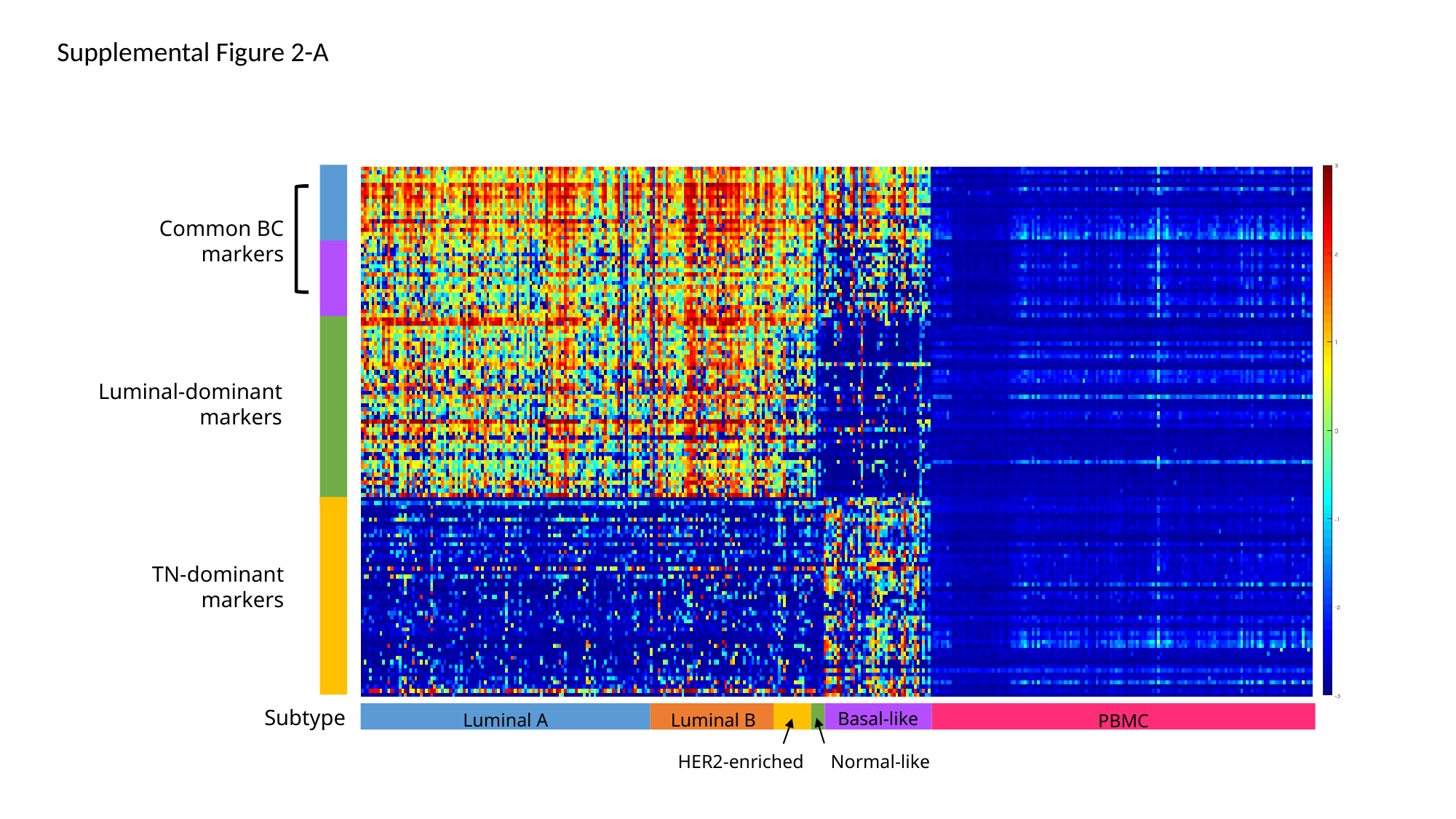

Supplemental Figure 2-A
Common BC
 markers
Luminal-dominant
markers
TN-dominant
markers
Subtype
Basal-like
Luminal A
Luminal B
PBMC
HER2-enriched
Normal-like

## Slide 3
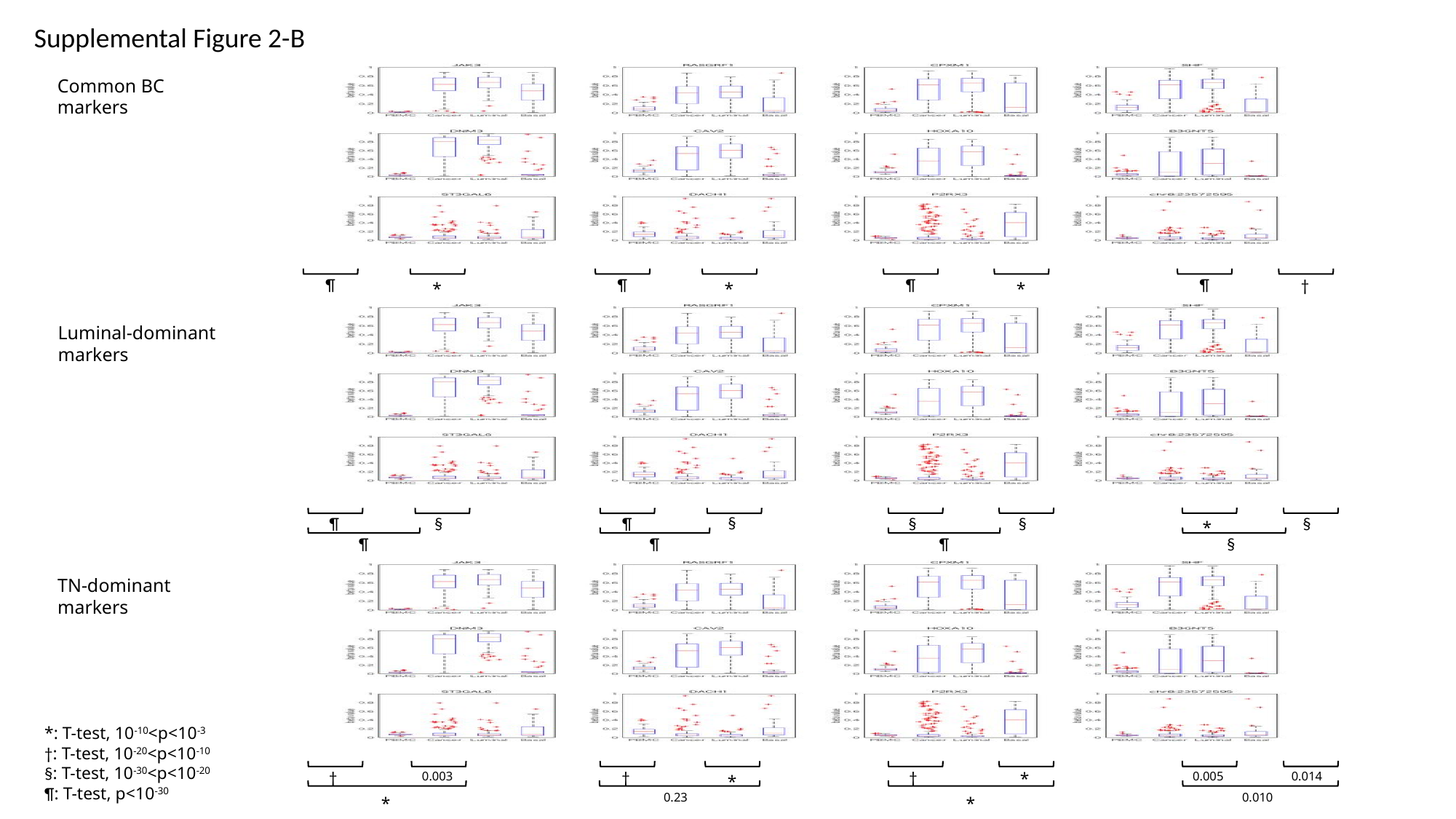

Supplemental Figure 2-B
Common BC
markers
¶
¶
¶
¶
†
*
*
*
Luminal-dominant
markers
§
¶
§
§
§
§
¶
*
¶
¶
¶
§
TN-dominant
markers
*: T-test, 10-10<p<10-3
†: T-test, 10-20<p<10-10
§: T-test, 10-30<p<10-20
¶: T-test, p<10-30
†
†
†
0.003
*
0.005
0.014
*
0.23
0.010
*
*

## Slide 4
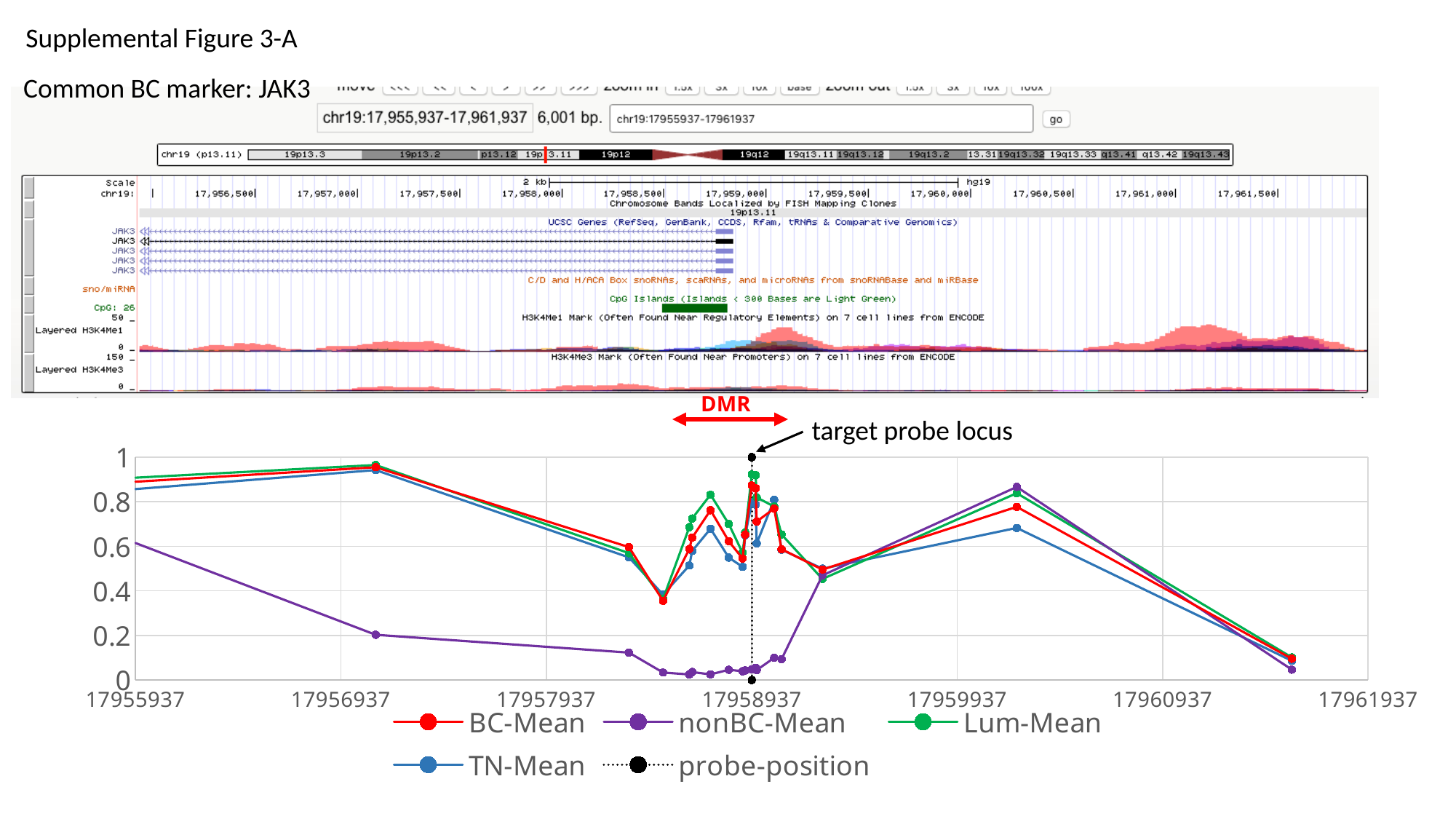

Supplemental Figure 3-A
Common BC marker: JAK3
DMR
target probe locus
### Chart
| Category | | | | | |
|---|---|---|---|---|---|

## Slide 5
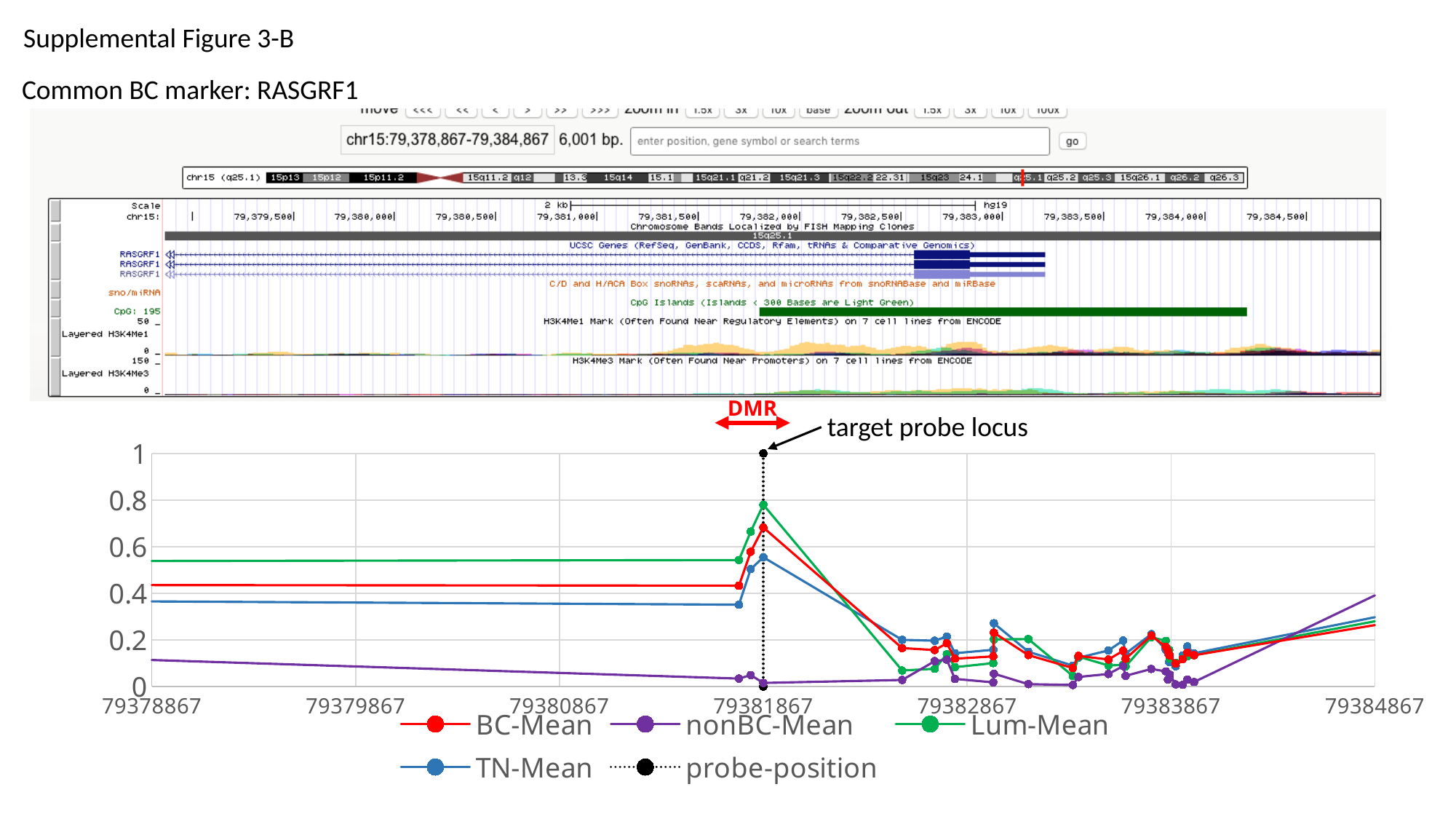

Supplemental Figure 3-B
Common BC marker: RASGRF1
DMR
target probe locus
### Chart
| Category | | | | | |
|---|---|---|---|---|---|

## Slide 6
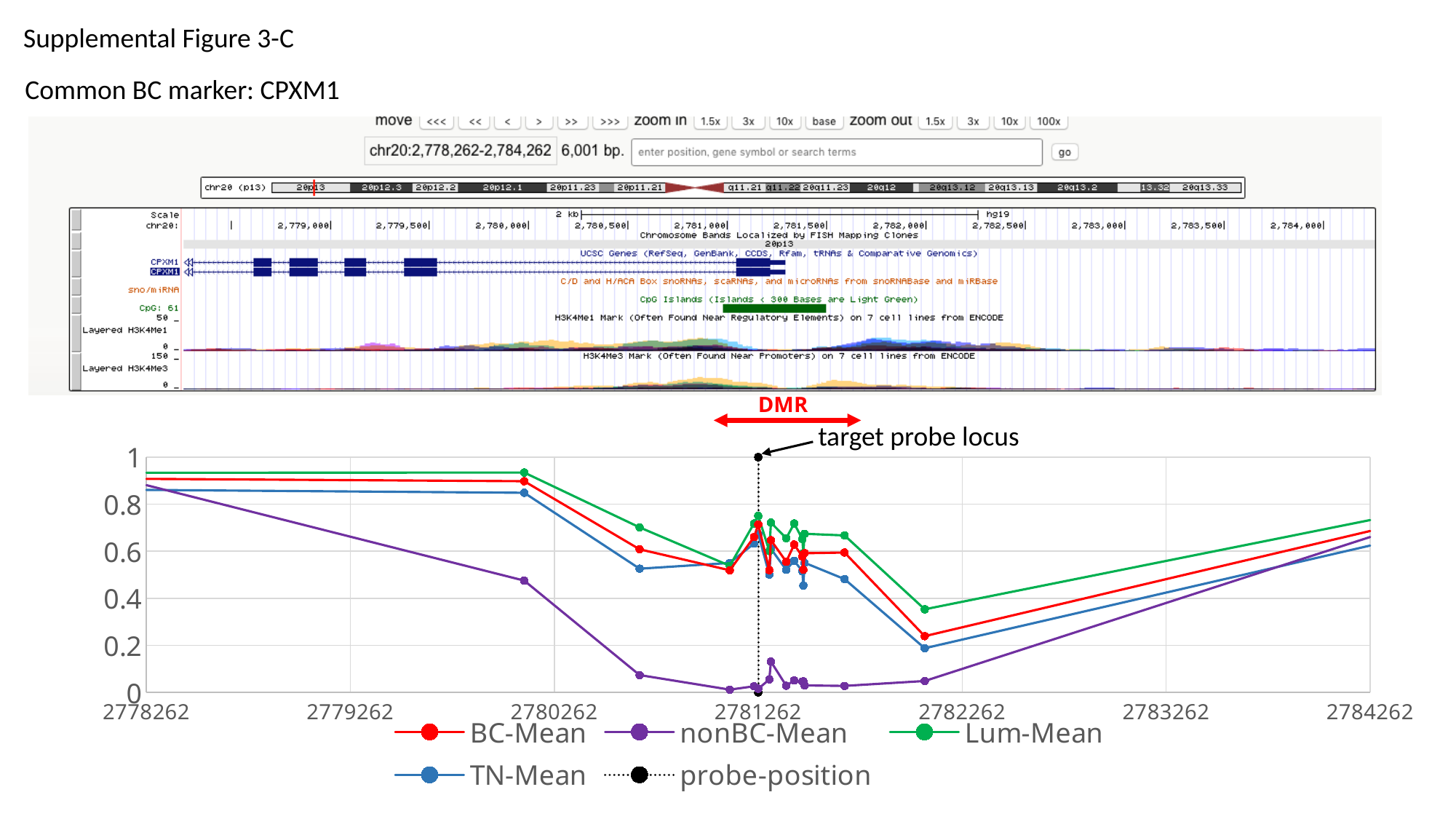

Supplemental Figure 3-C
Common BC marker: CPXM1
DMR
target probe locus
### Chart
| Category | | | | | |
|---|---|---|---|---|---|

## Slide 7
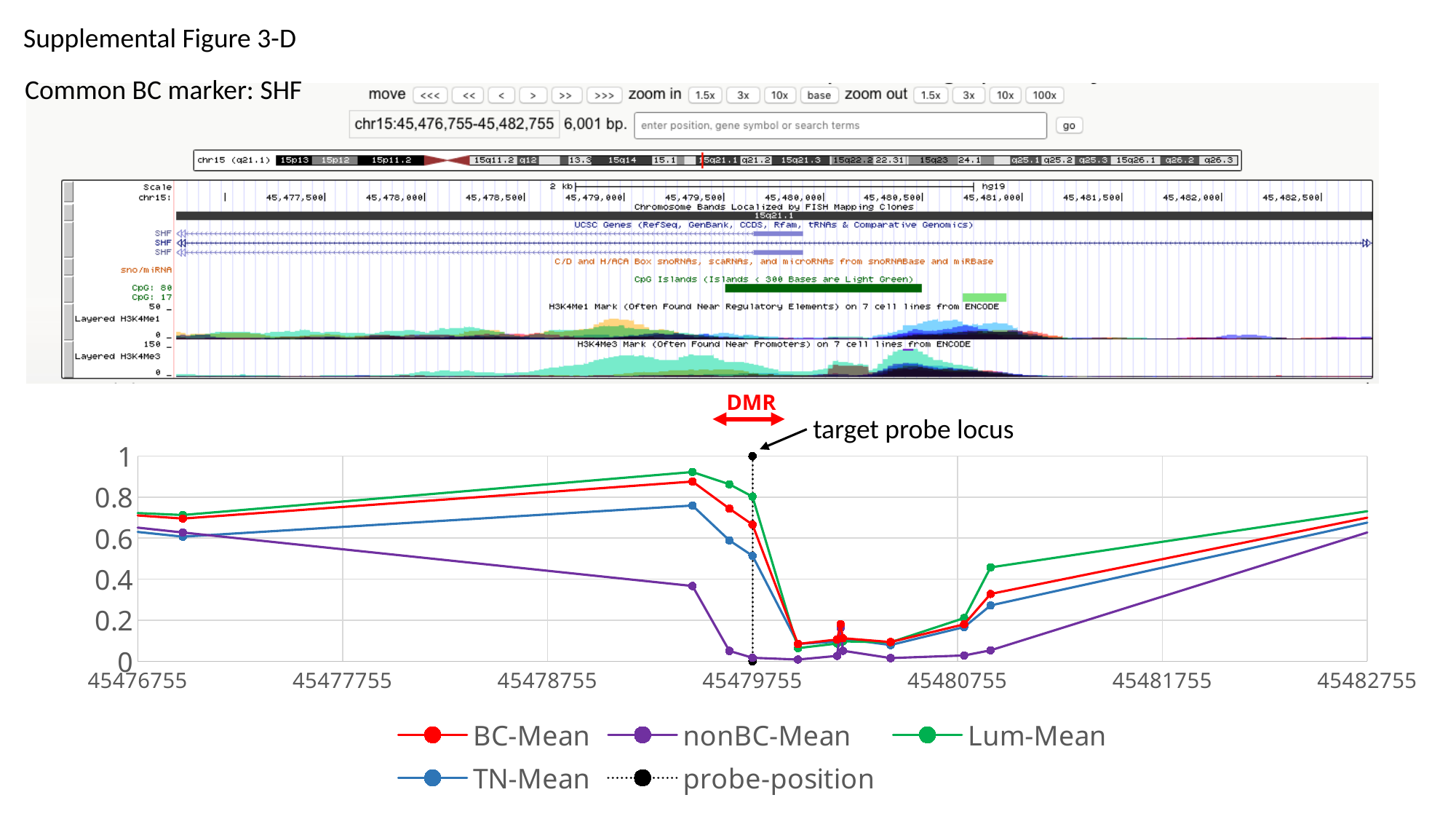

Supplemental Figure 3-D
Common BC marker: SHF
DMR
target probe locus
### Chart
| Category | | | | | |
|---|---|---|---|---|---|

## Slide 8
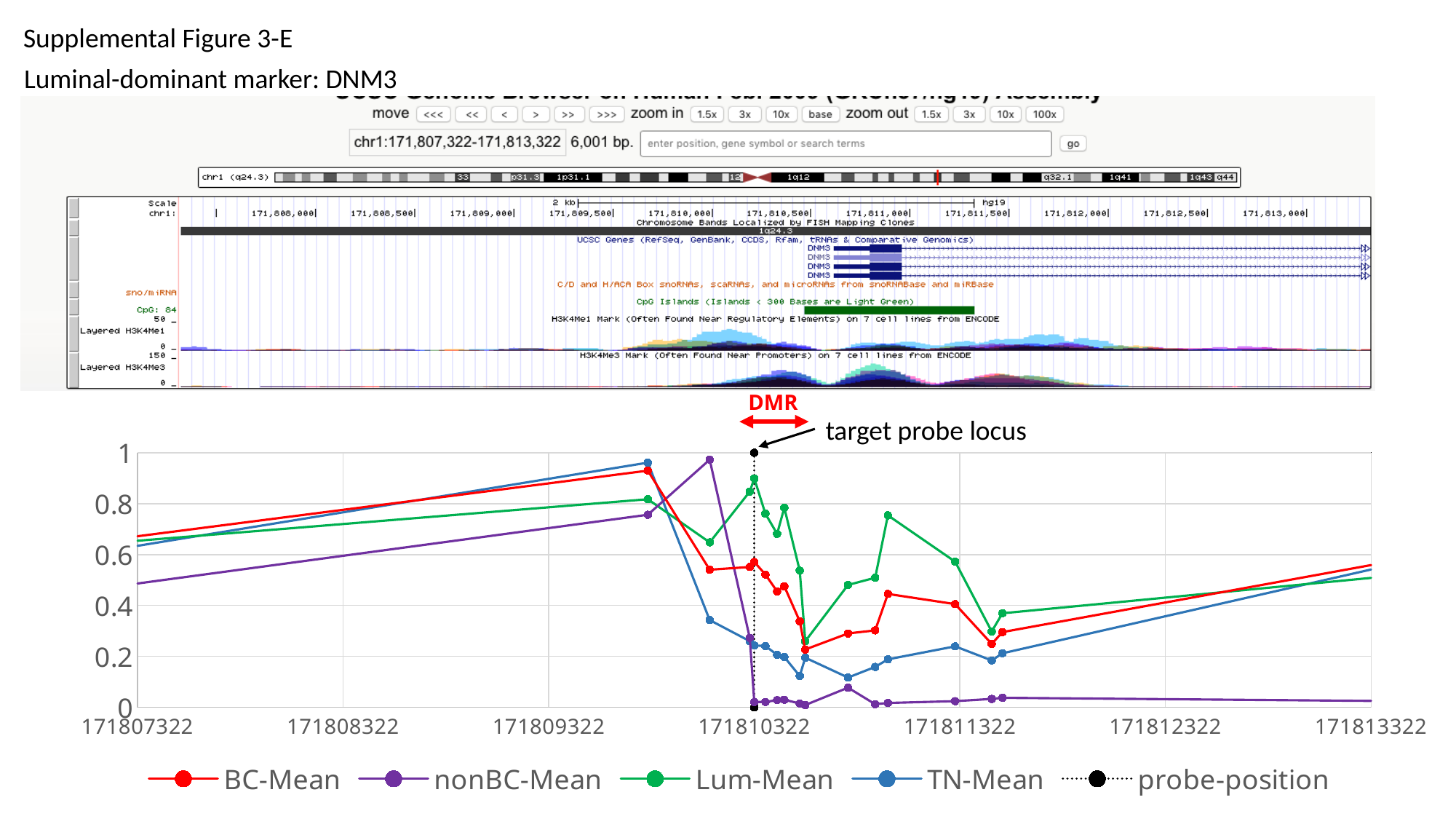

Supplemental Figure 3-E
Luminal-dominant marker: DNM3
DMR
target probe locus
### Chart
| Category | | | | | |
|---|---|---|---|---|---|

## Slide 9
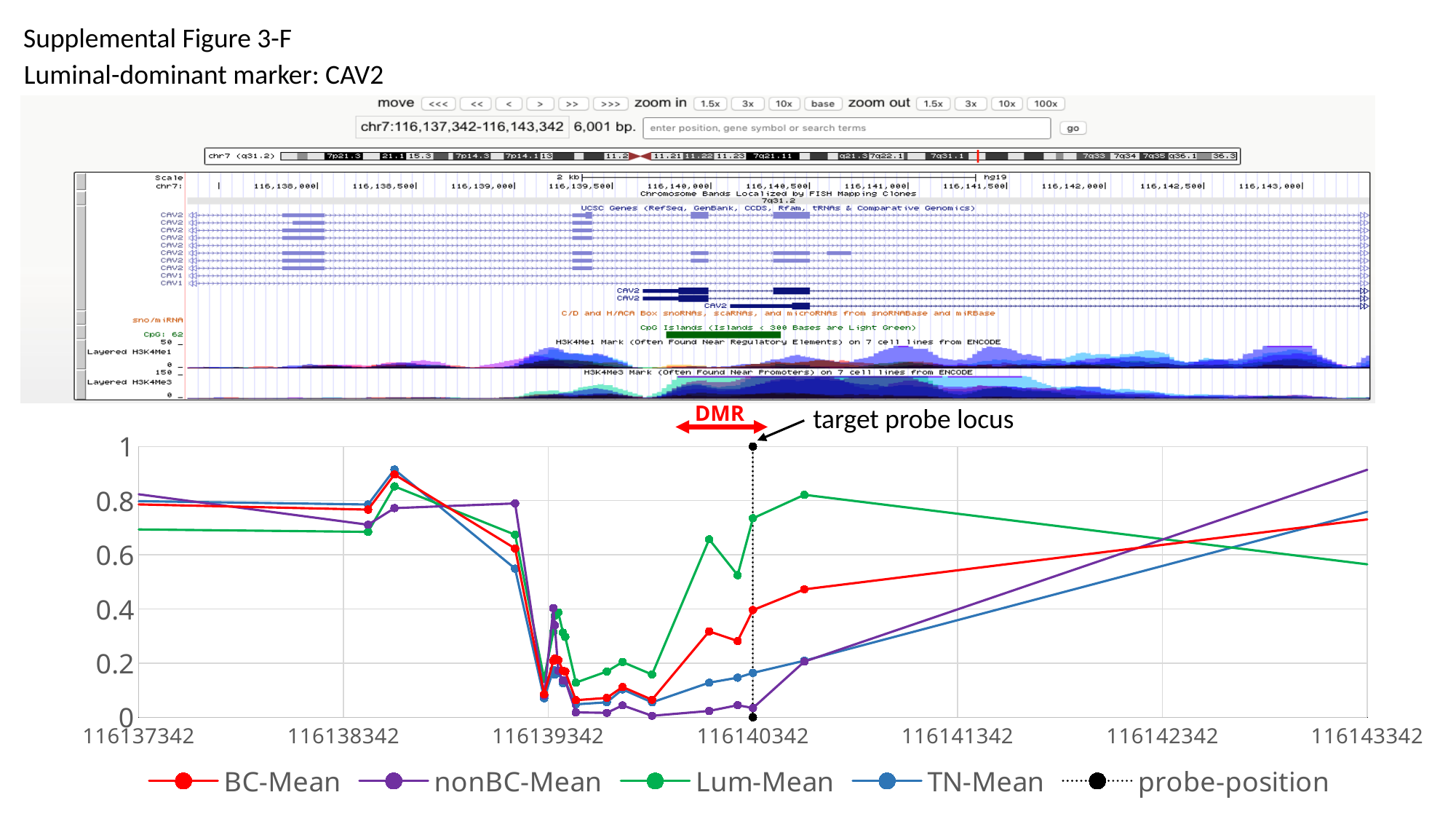

Supplemental Figure 3-F
Luminal-dominant marker: CAV2
DMR
target probe locus
### Chart
| Category | | | | | |
|---|---|---|---|---|---|

## Slide 10
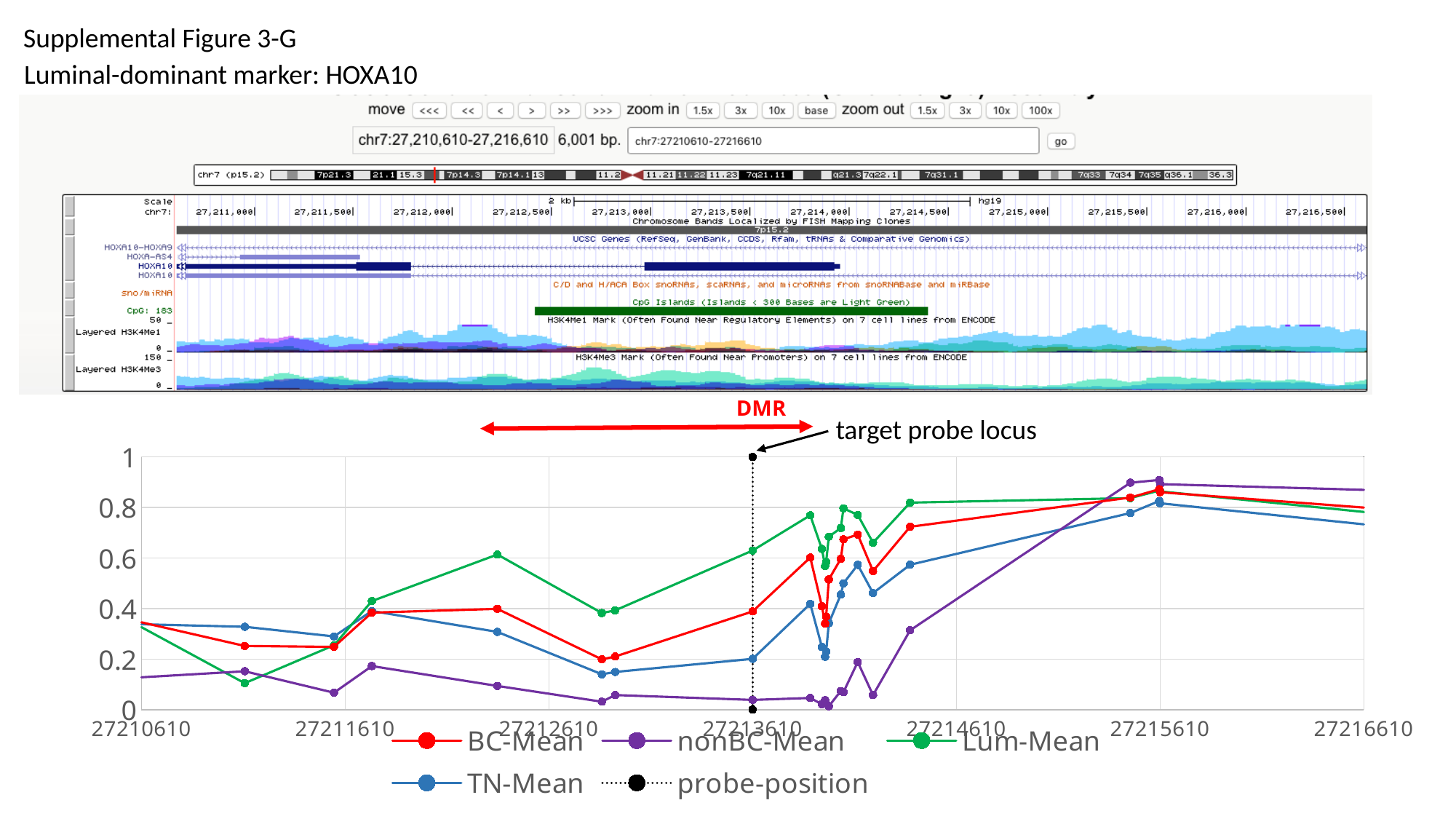

Supplemental Figure 3-G
Luminal-dominant marker: HOXA10
DMR
target probe locus
### Chart
| Category | | | | | |
|---|---|---|---|---|---|

## Slide 11
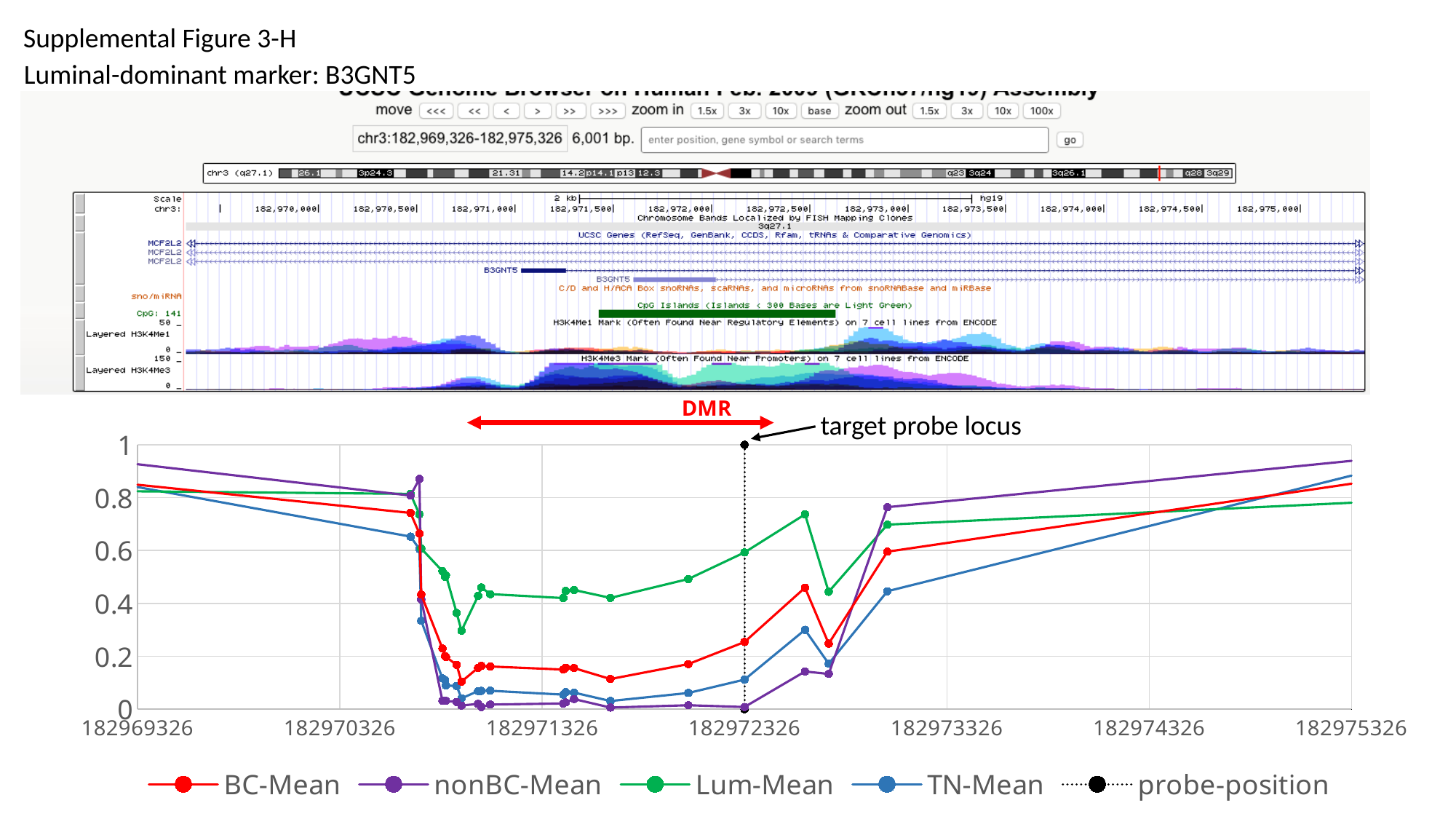

Supplemental Figure 3-H
Luminal-dominant marker: B3GNT5
DMR
target probe locus
### Chart
| Category | | | | | |
|---|---|---|---|---|---|

## Slide 12
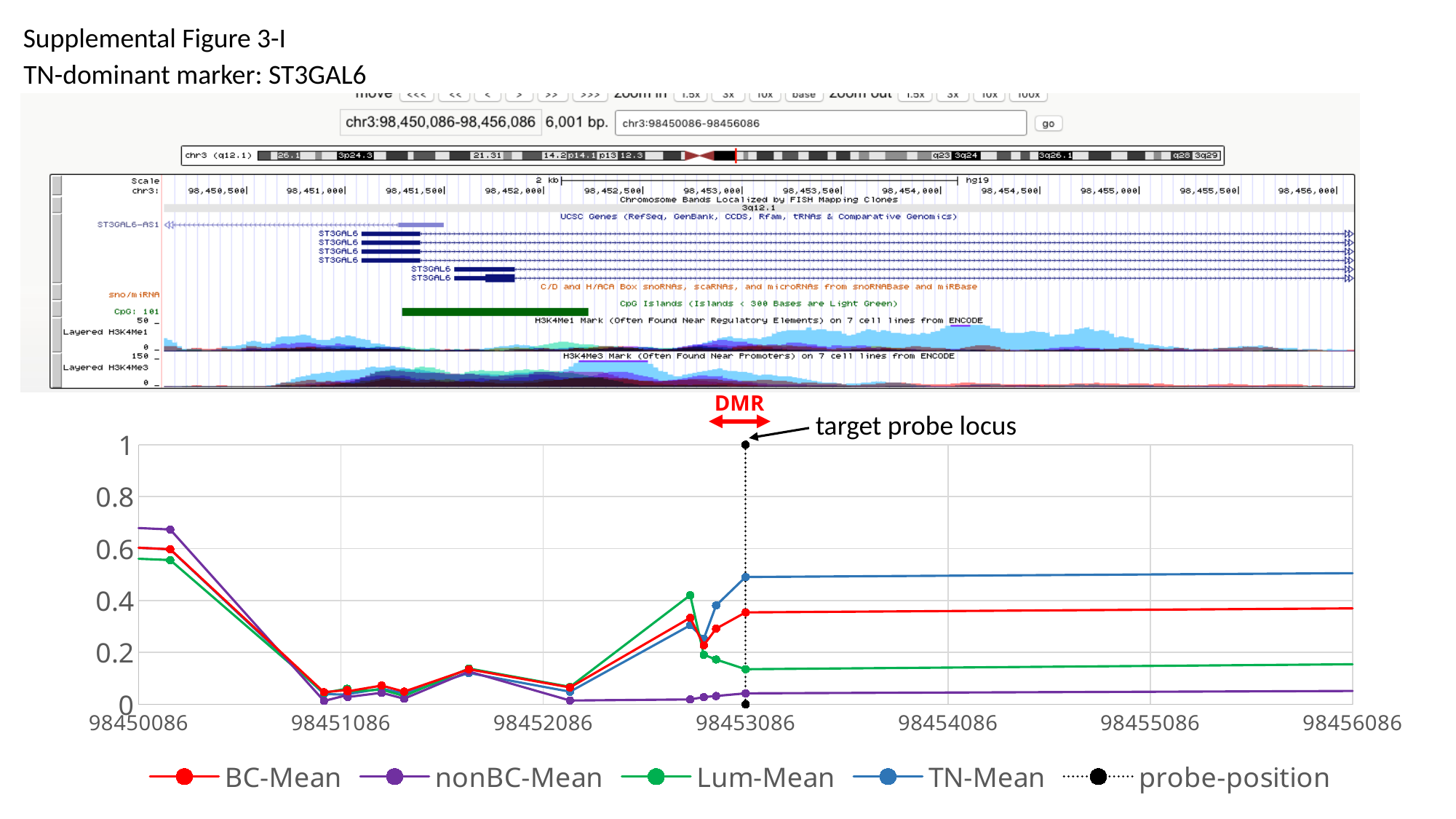

Supplemental Figure 3-I
TN-dominant marker: ST3GAL6
DMR
target probe locus
### Chart
| Category | | | | | |
|---|---|---|---|---|---|

## Slide 13
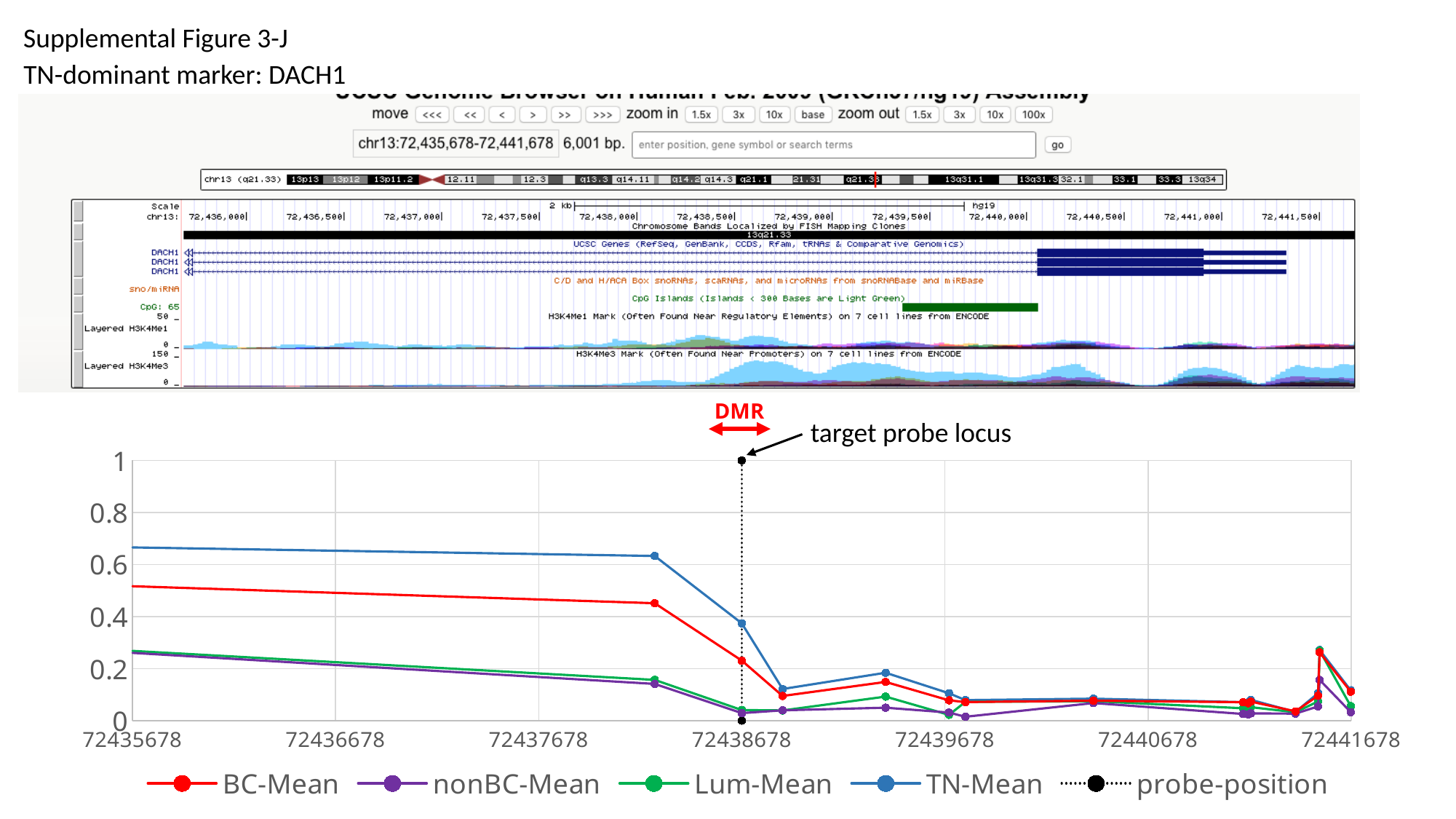

Supplemental Figure 3-J
TN-dominant marker: DACH1
DMR
target probe locus
### Chart
| Category | | | | | |
|---|---|---|---|---|---|

## Slide 14
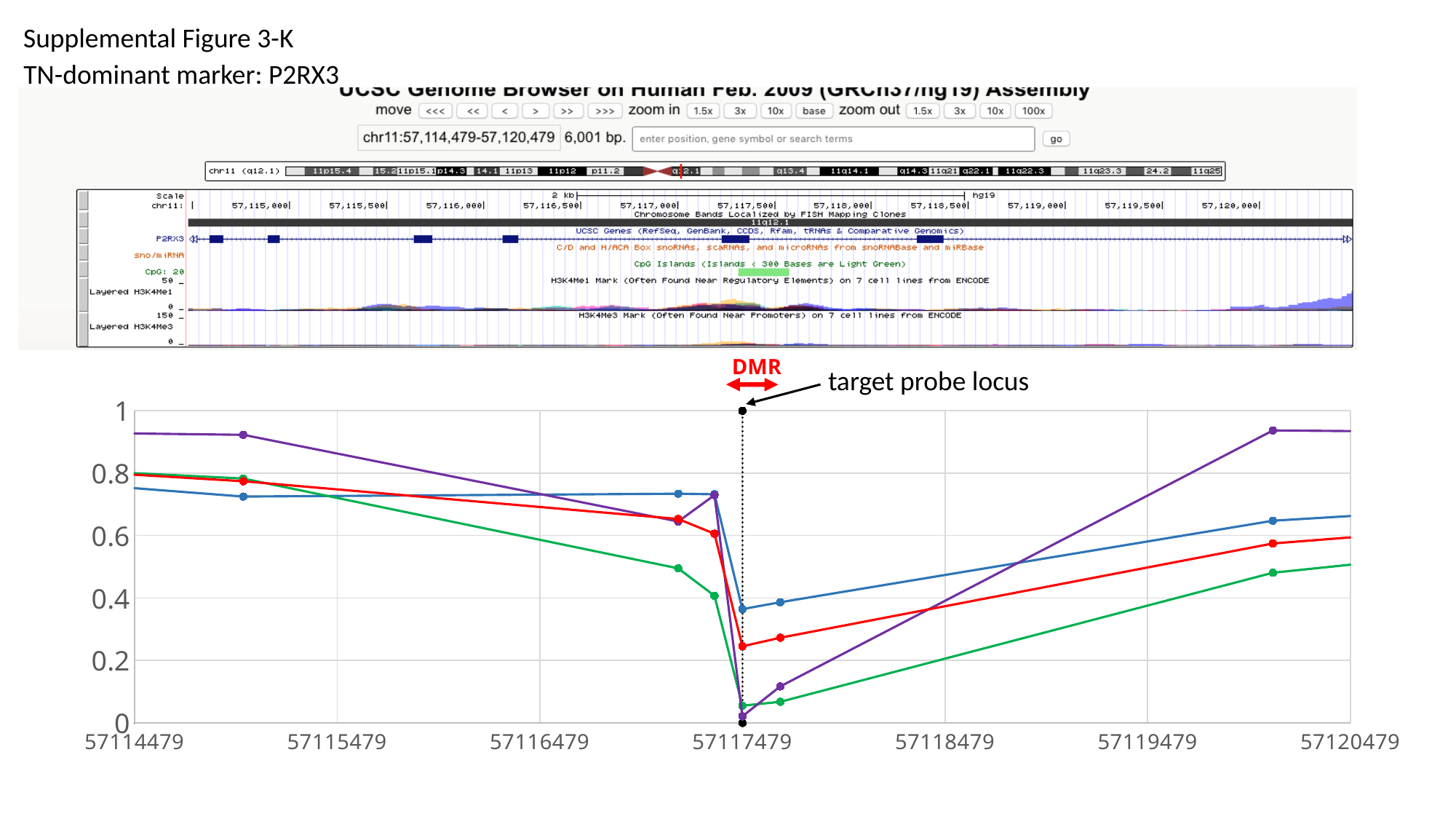

Supplemental Figure 3-K
TN-dominant marker: P2RX3
DMR
target probe locus
### Chart
| Category | | | | | |
|---|---|---|---|---|---|

## Slide 15
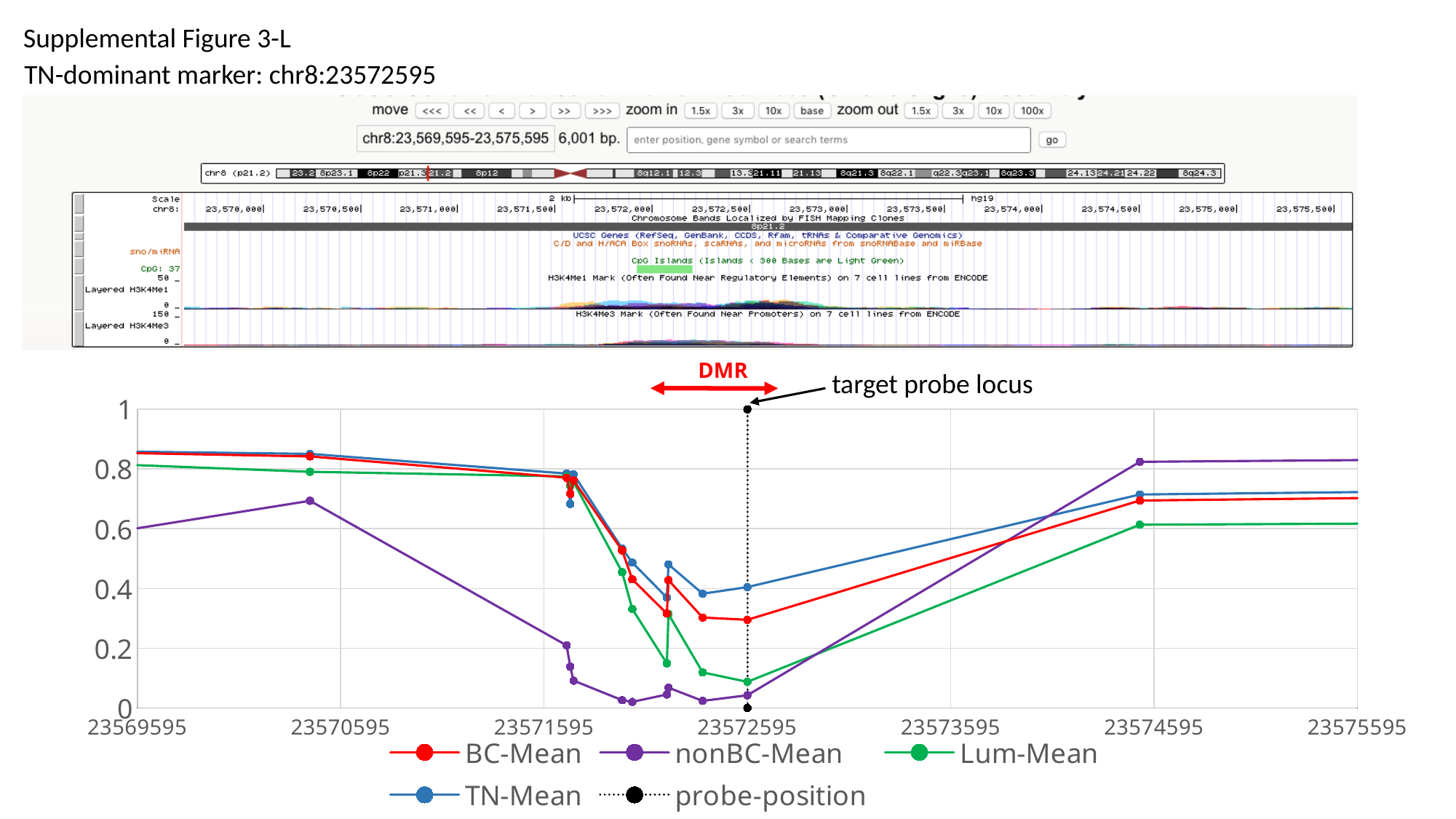

Supplemental Figure 3-L
TN-dominant marker: chr8:23572595
DMR
target probe locus
### Chart
| Category | | | | | |
|---|---|---|---|---|---|

## Slide 16
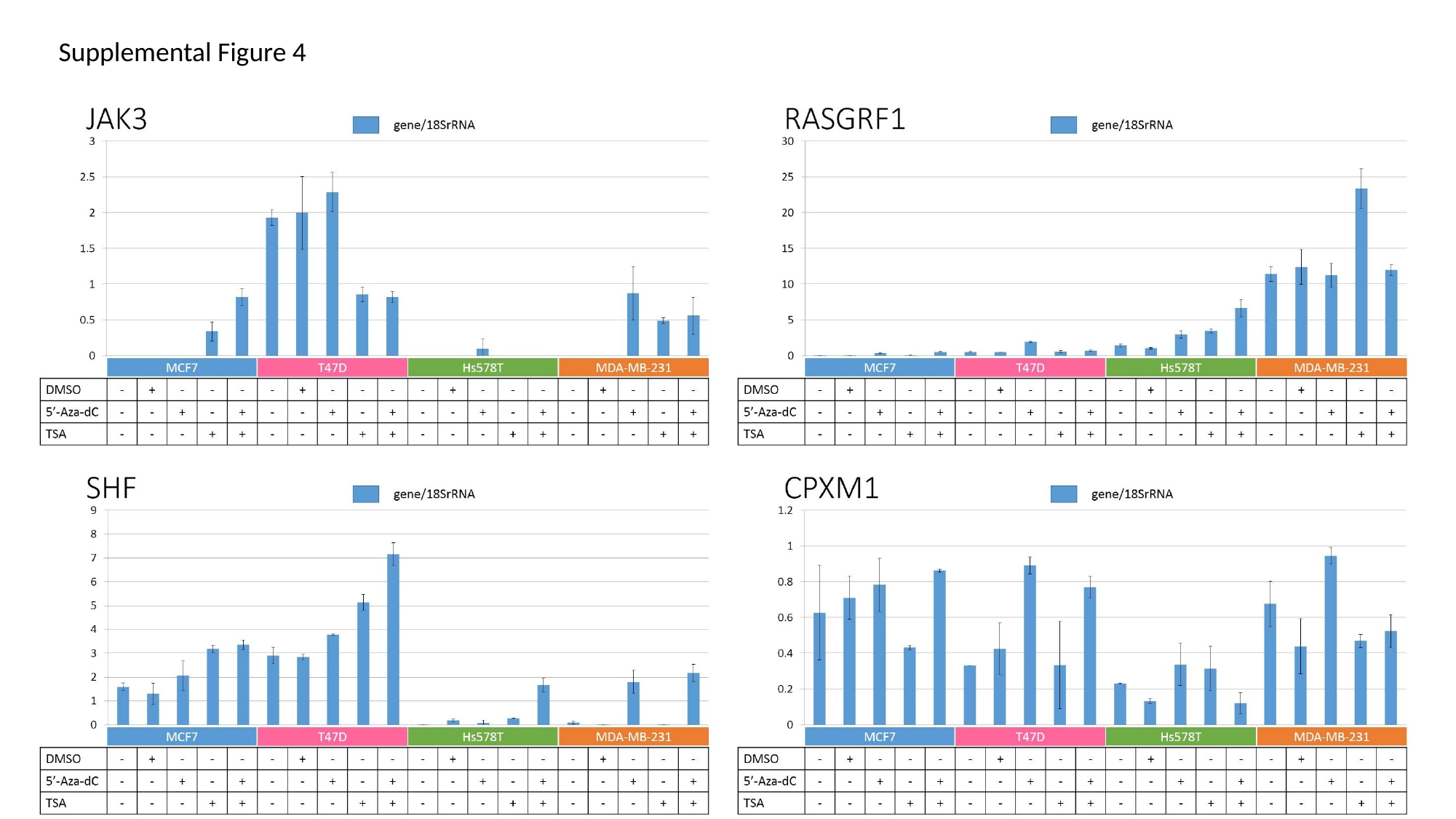

Supplemental Figure 4

## Slide 17
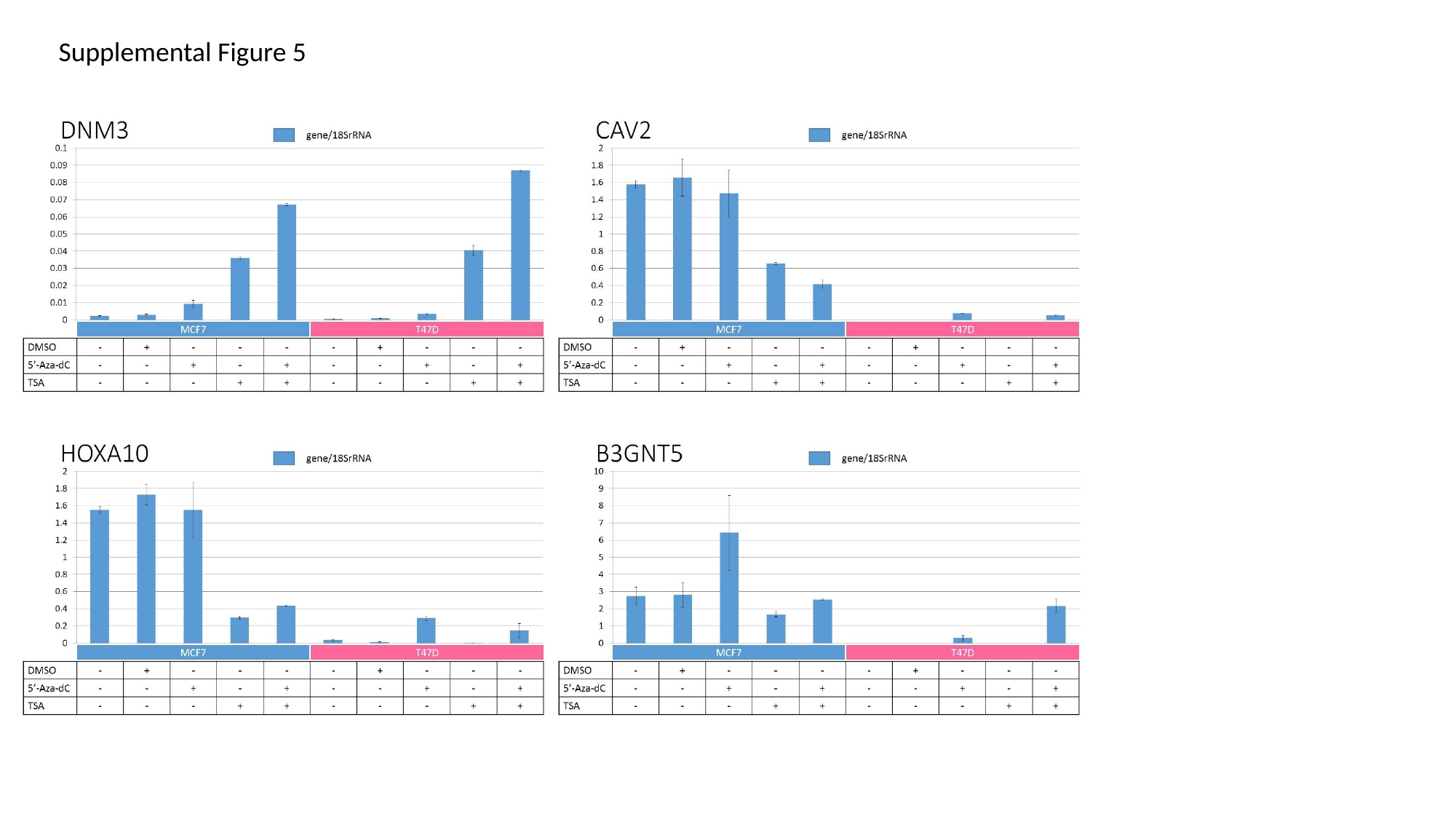

Supplemental Figure 5

## Slide 18
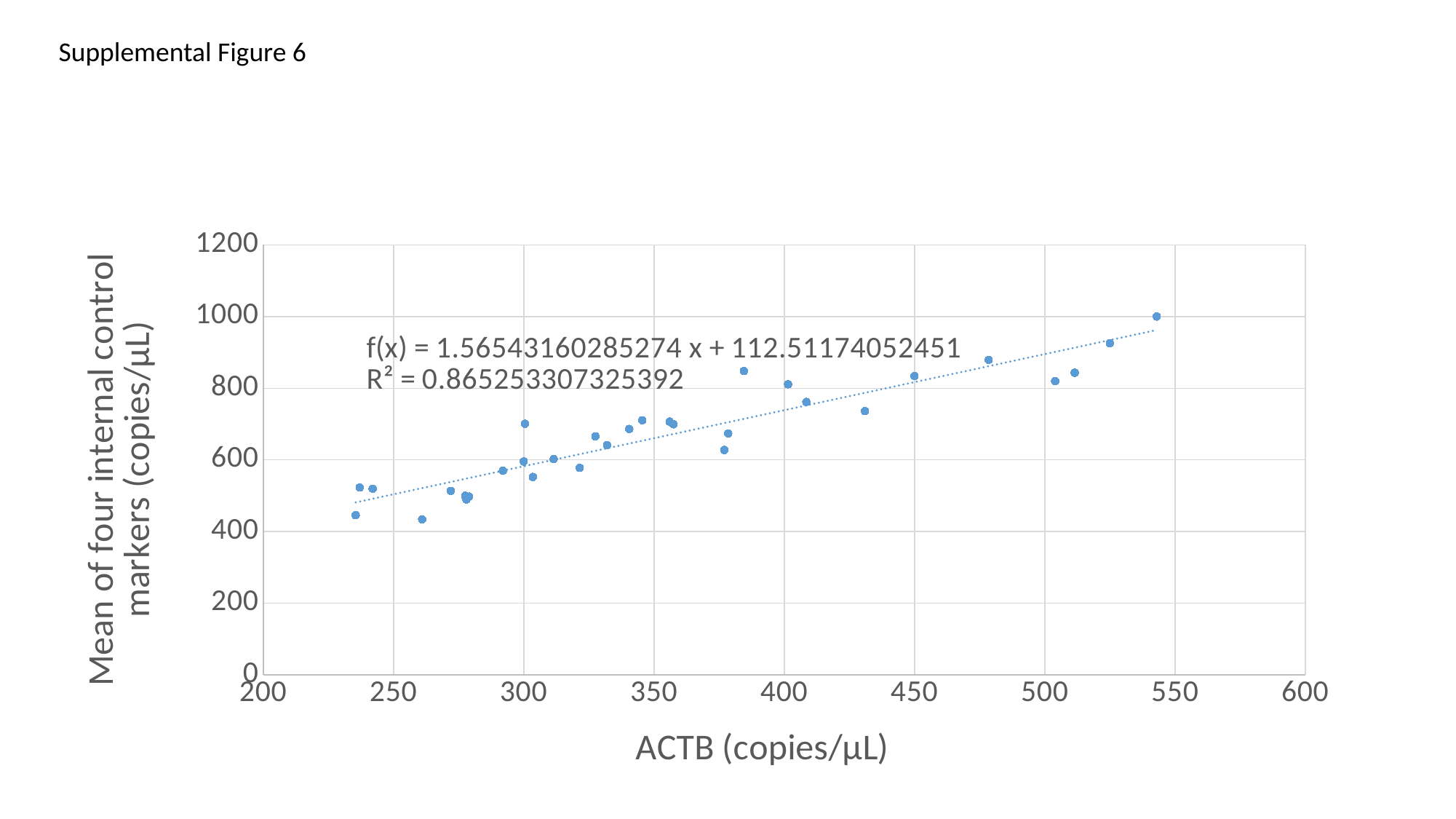

Supplemental Figure 6
### Chart
| Category | 4genes |
|---|---|

## Slide 19
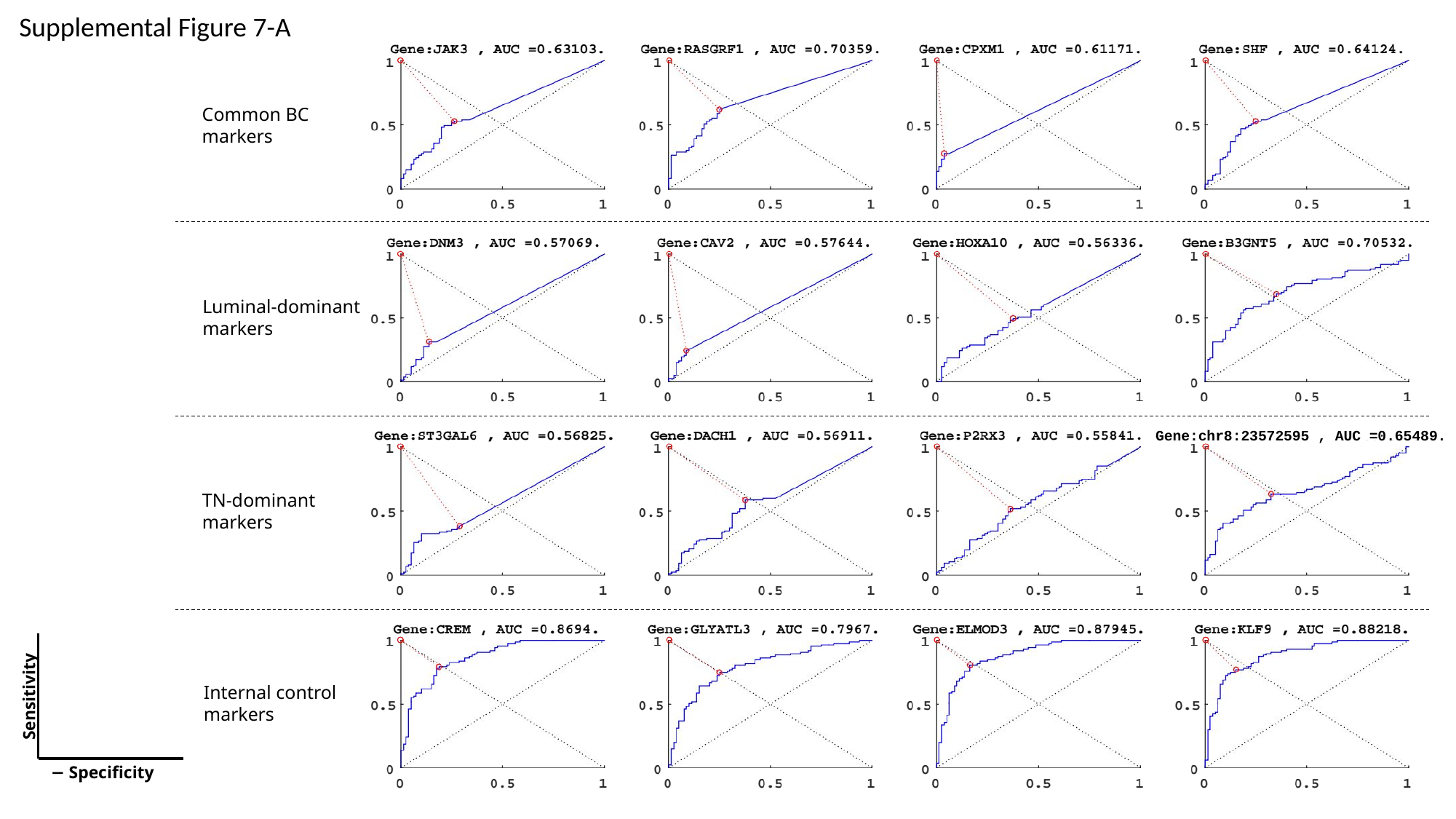

Supplemental Figure 7-A
Common BC
markers
Luminal-dominant
markers
Gene:chr8:23572595 , AUC =0.65489.
TN-dominant
markers
Sensitivity
１−Specificity
Internal control
markers

## Slide 20
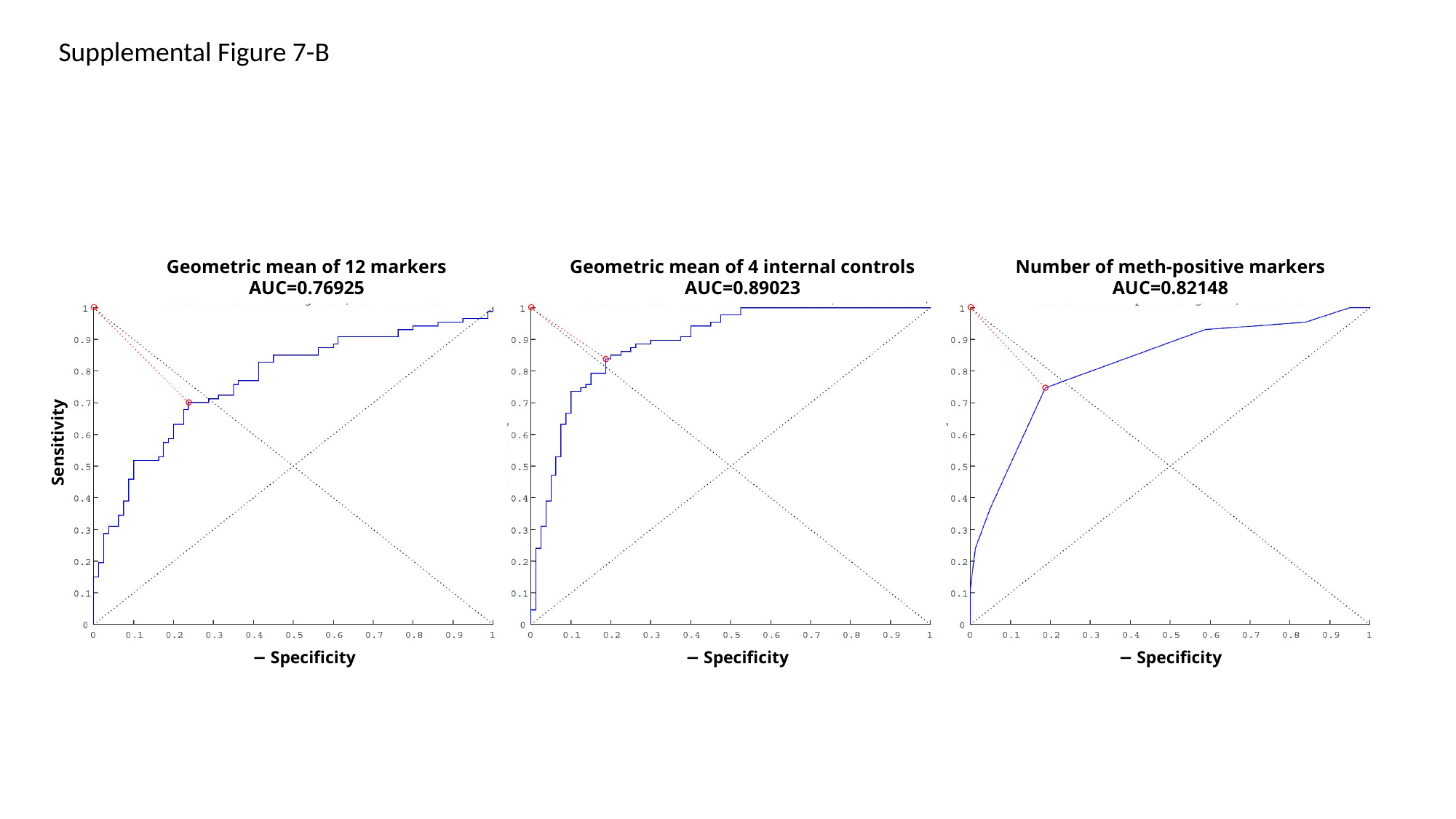

Geometric mean of 12 markers
AUC=0.76925
Supplemental Figure 7-B
Number of meth-positive markers
AUC=0.82148
Geometric mean of 4 internal controls
AUC=0.89023
Geometric mean of 12 markers
AUC=0.76925
Sensitivity
１−Specificity
１−Specificity
１−Specificity

## Slide 21
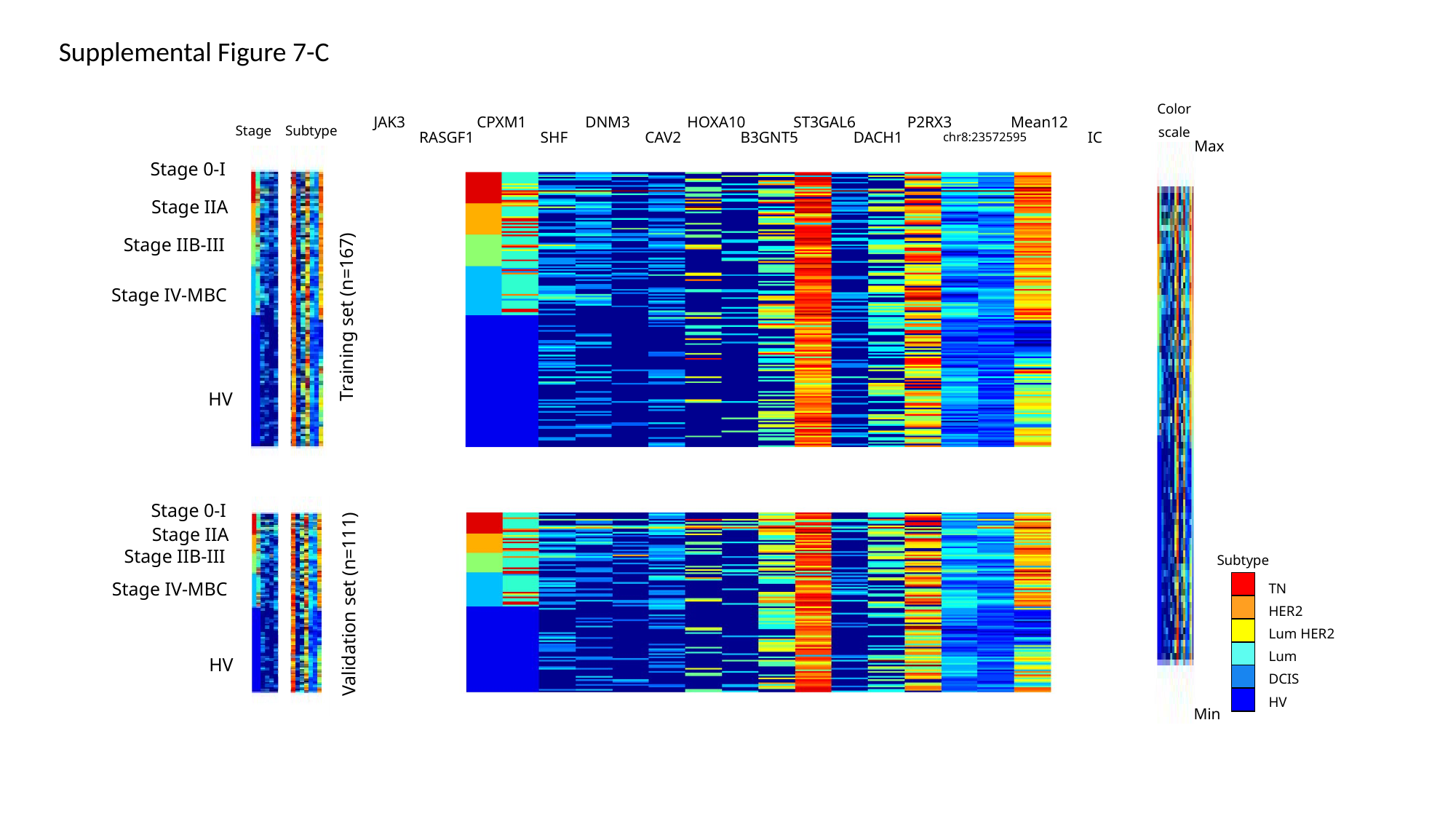

Supplemental Figure 7-C
Color
scale
JAK3
CPXM1
DNM3
HOXA10
ST3GAL6
P2RX3
Mean12
Subtype
Stage
RASGF1
SHF
CAV2
B3GNT5
DACH1
IC
chr8:23572595
Max
Stage 0-I
Stage IIA
Stage IIB-III
Stage IV-MBC
Training set (n=167)
HV
Stage 0-I
Stage IIA
Stage IIB-III
Subtype
TN
HER2
Lum HER2
Lum
DCIS
HV
Stage IV-MBC
Validation set (n=111)
HV
Min

## Slide 22
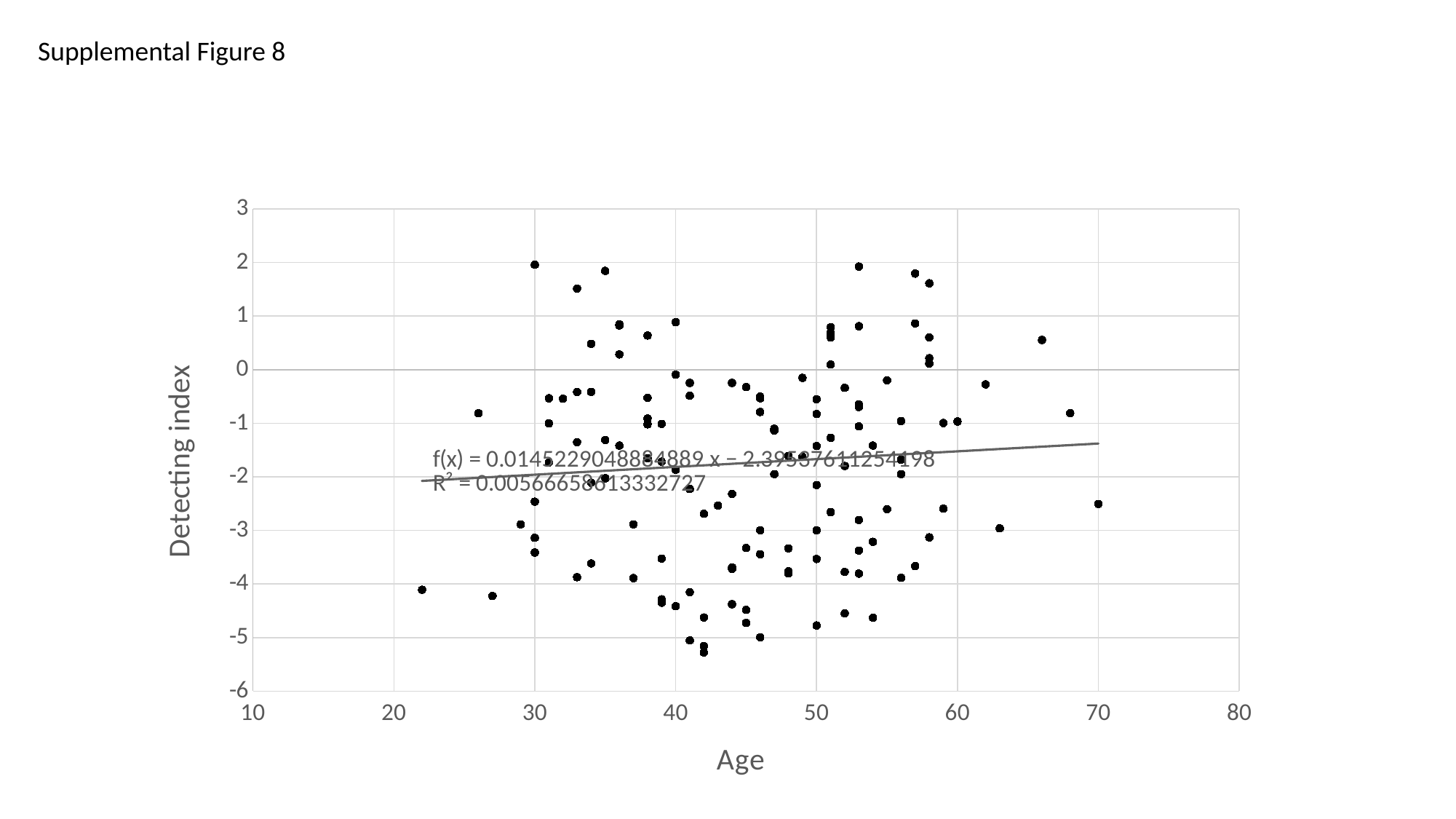

# Supplemental Figure 8
### Chart
| Category | prediction |
|---|---|

## Slide 23
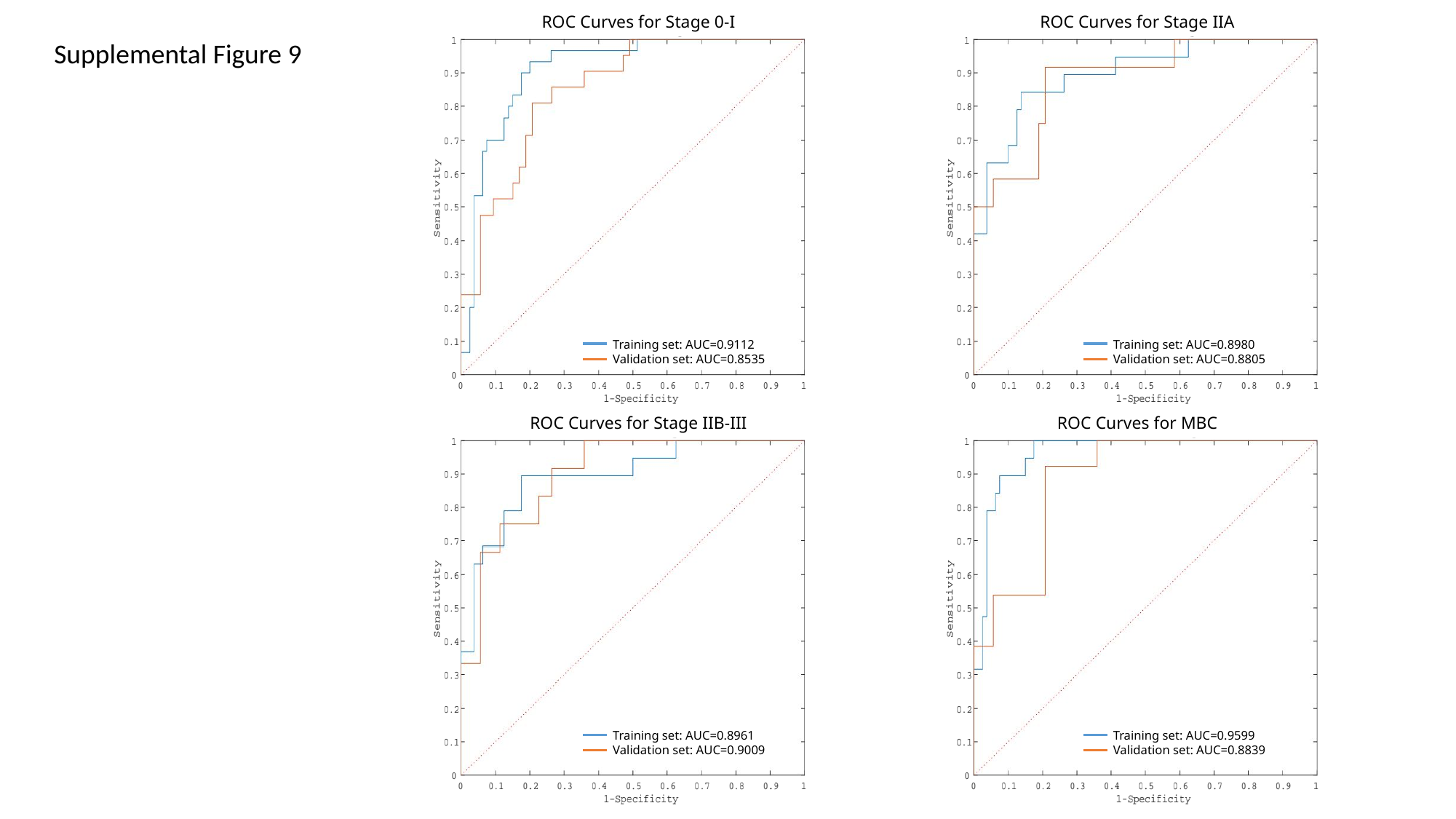

ROC Curves for Stage 0-I
ROC Curves for Stage IIA
Supplemental Figure 9
Training set: AUC=0.9112
Validation set: AUC=0.8535
Training set: AUC=0.8980
Validation set: AUC=0.8805
ROC Curves for Stage IIB-III
ROC Curves for MBC
Training set: AUC=0.8961
Validation set: AUC=0.9009
Training set: AUC=0.9599
Validation set: AUC=0.8839

## Slide 24
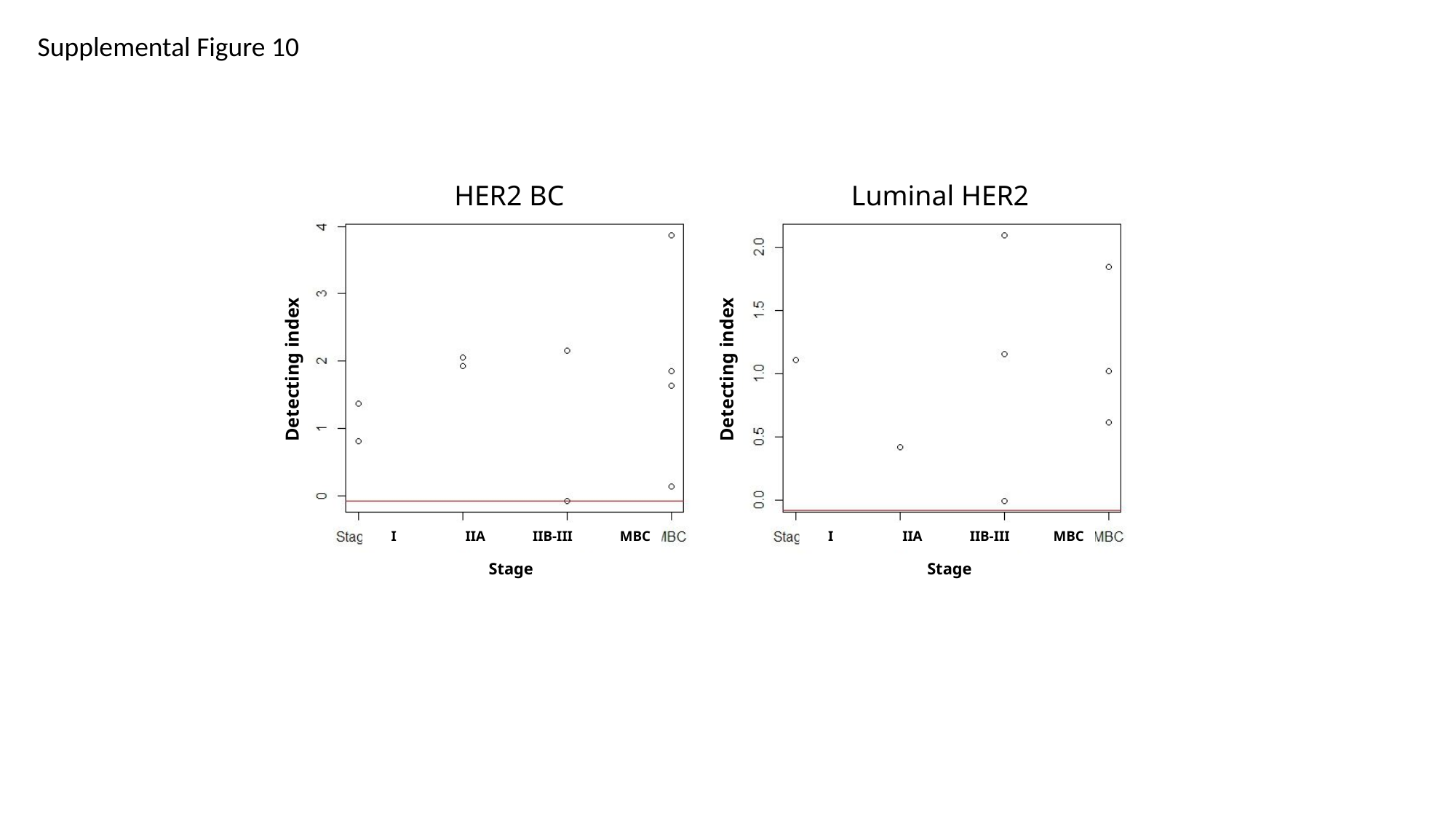

Supplemental Figure 10
HER2 BC
Luminal HER2
Detecting index
Detecting index
 I IIA IIB-III MBC
 I IIA IIB-III MBC
Stage
Stage

## Slide 25
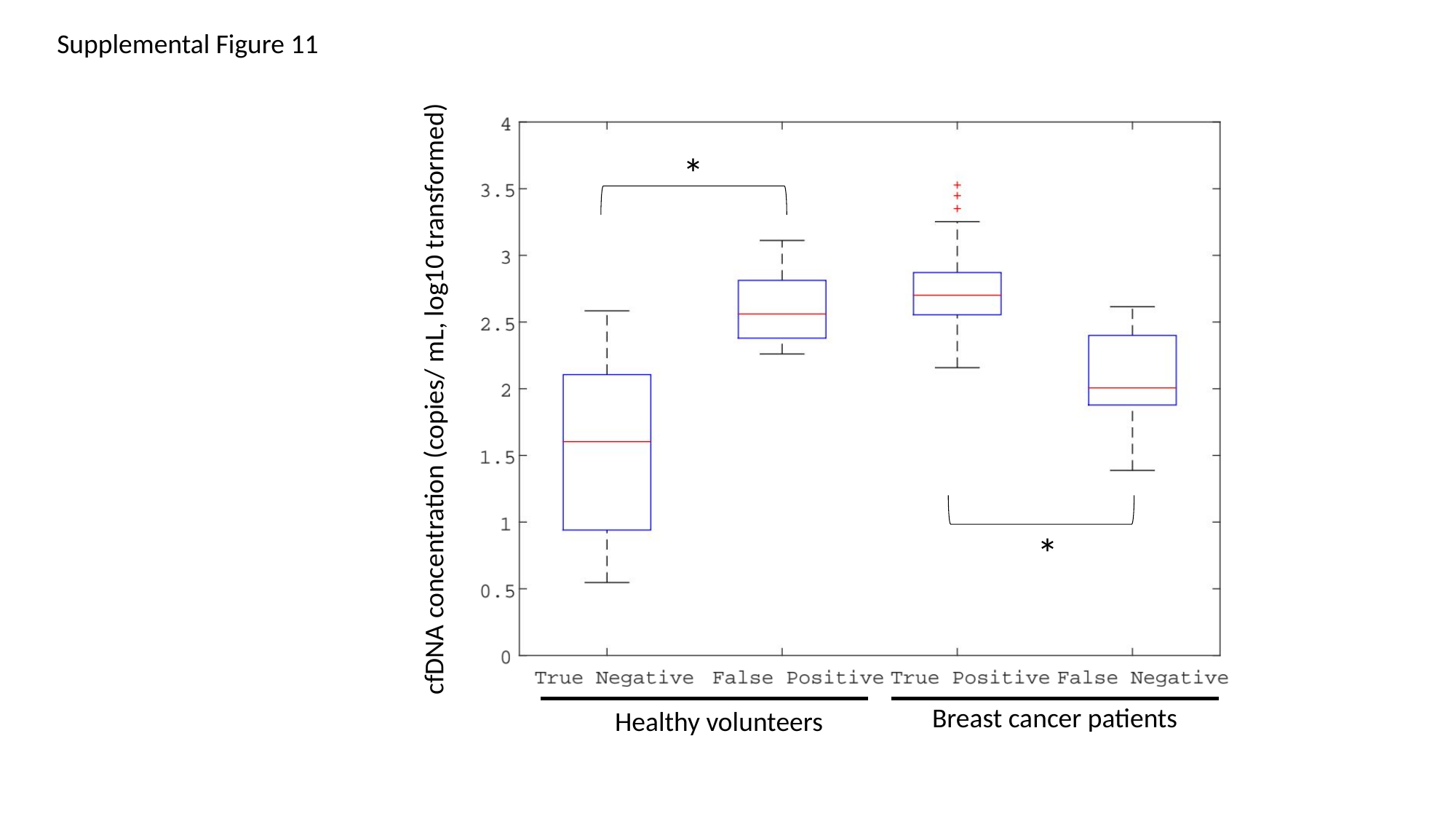

Supplemental Figure 11
*
cfDNA concentration (copies/ mL, log10 transformed)
*
Breast cancer patients
Healthy volunteers

## Slide 26
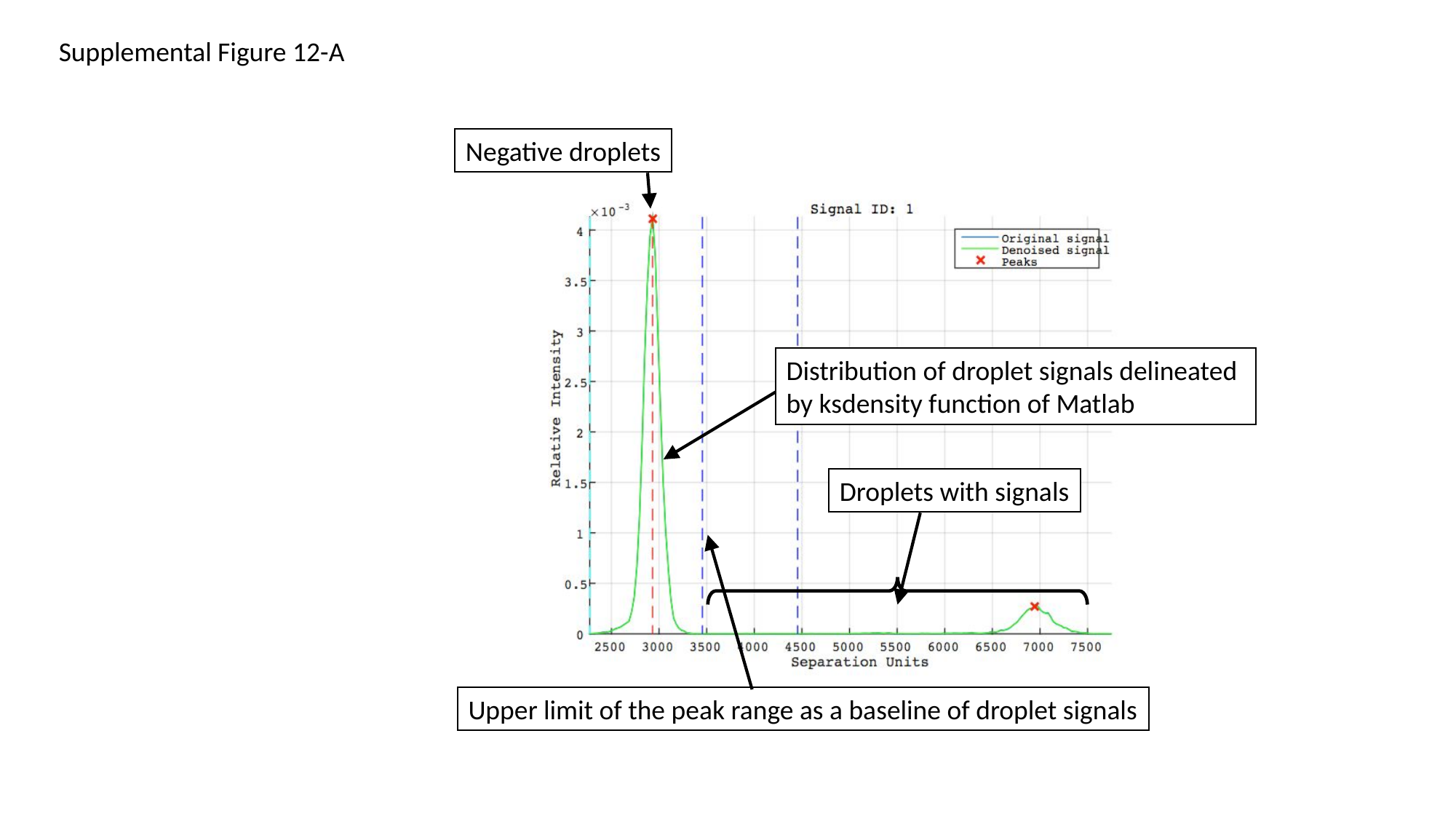

Supplemental Figure 12-A
Negative droplets
Distribution of droplet signals delineated by ksdensity function of Matlab
Droplets with signals
Upper limit of the peak range as a baseline of droplet signals

## Slide 27
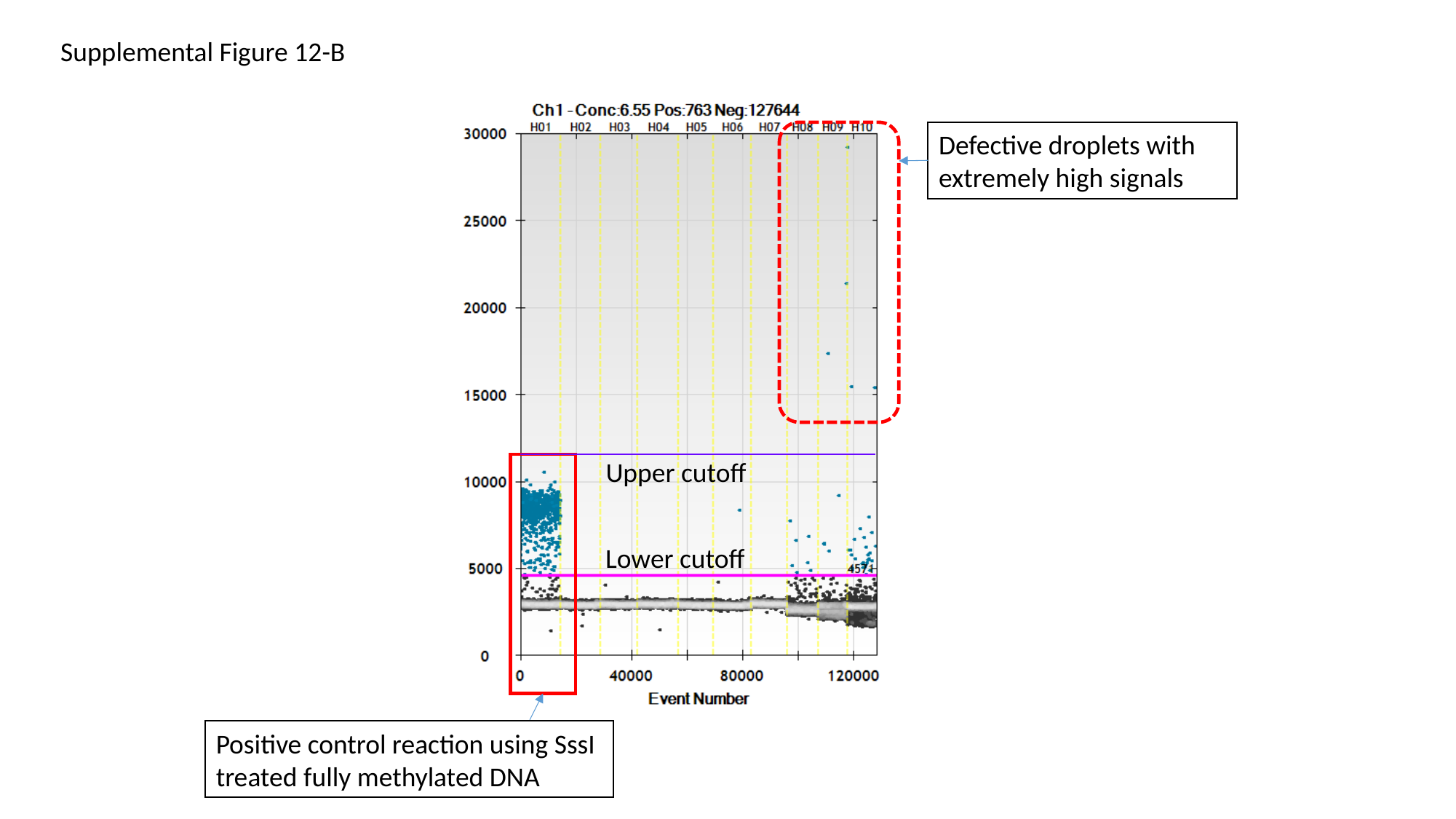

Supplemental Figure 12-B
Defective droplets with extremely high signals
Upper cutoff
Lower cutoff
Positive control reaction using SssI treated fully methylated DNA

## Slide 28
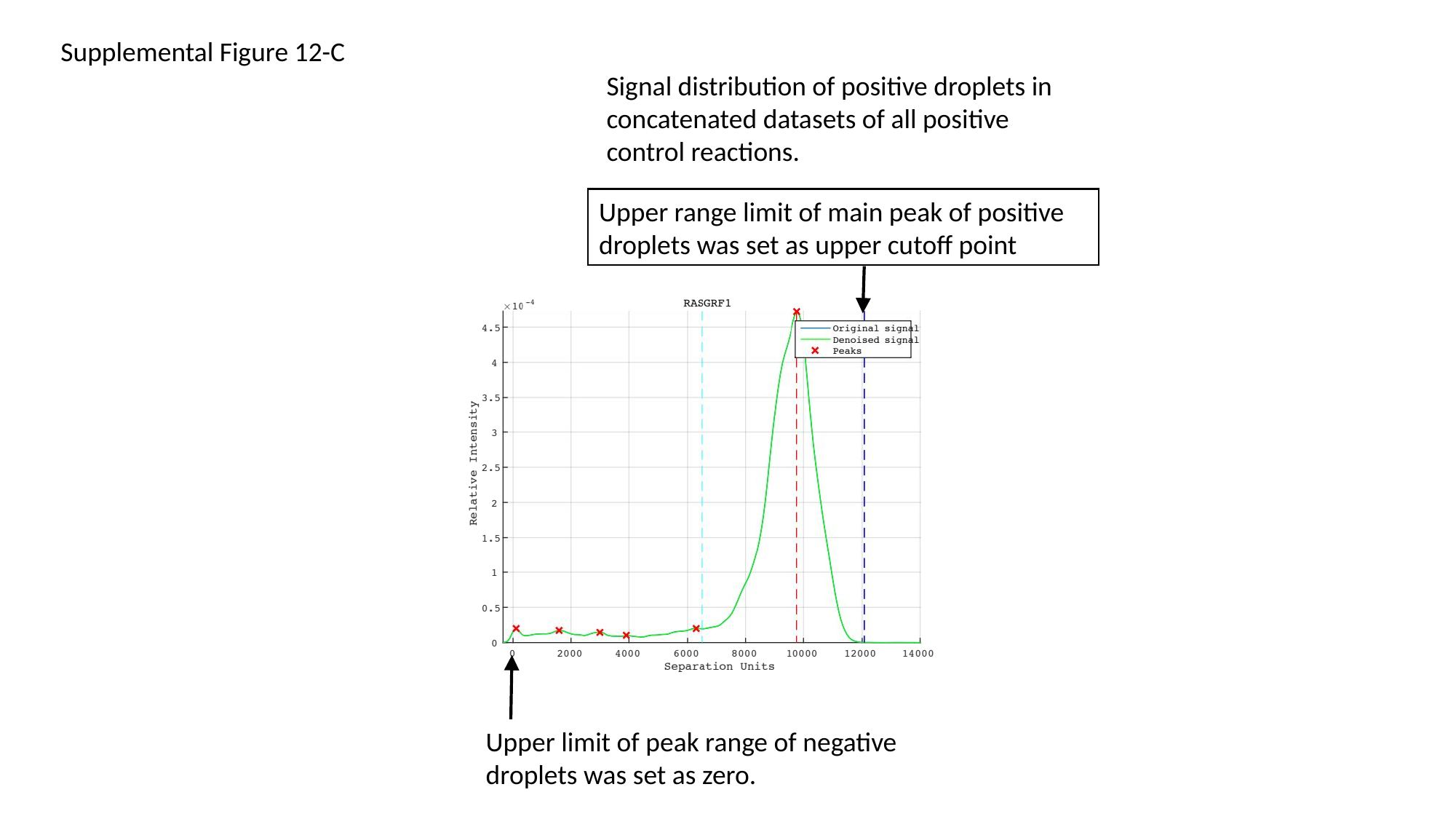

Supplemental Figure 12-C
Signal distribution of positive droplets in concatenated datasets of all positive control reactions.
Upper range limit of main peak of positive droplets was set as upper cutoff point
Upper limit of peak range of negative droplets was set as zero.

## Slide 29
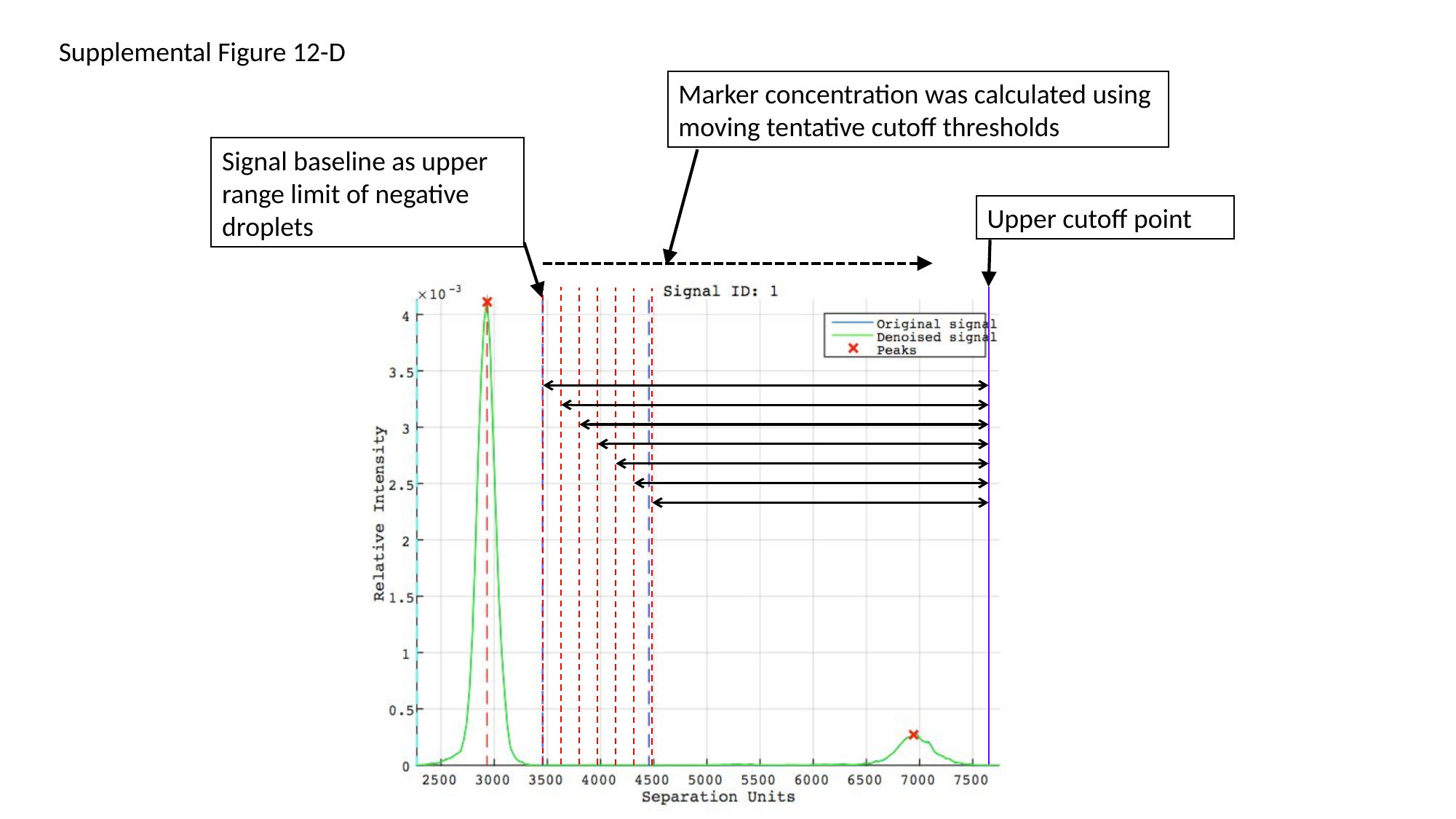

Supplemental Figure 12-D
Marker concentration was calculated using
moving tentative cutoff thresholds
Signal baseline as upper range limit of negative droplets
Upper cutoff point

## Slide 30
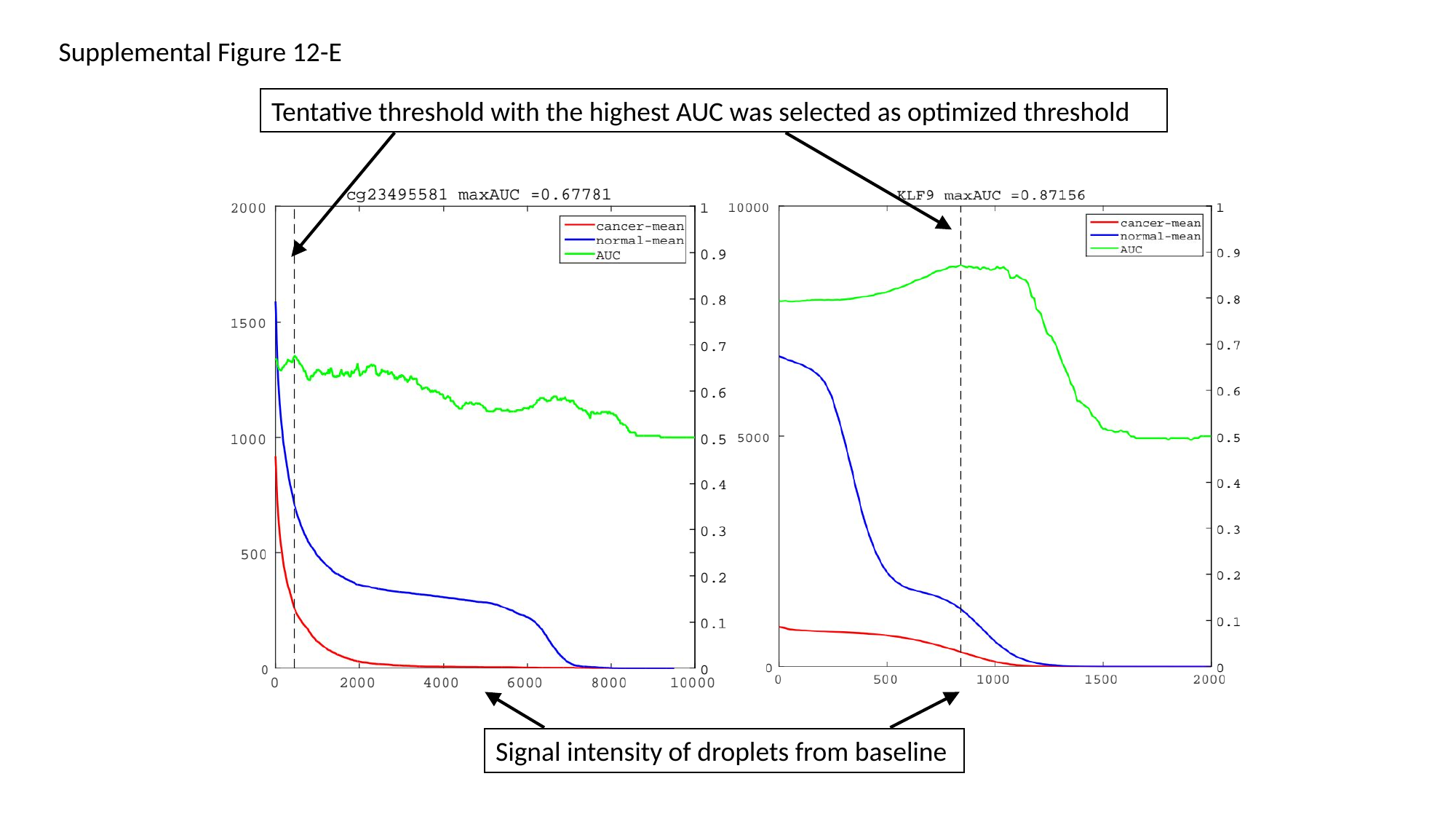

Supplemental Figure 12-E
Tentative threshold with the highest AUC was selected as optimized threshold
Signal intensity of droplets from baseline
